# Common and distinct equity preferences in children and adults

**DOI:** 10.3389/fpsyg.2024.1330024

**Published:** 2024-02-14

**Authors:** Han Xu, Lanxin Luo, Ruida Zhu, Yue Zhao, Luansu Zhang, Yaqi Zhang, Chunliang Feng, Qing Guan

**Affiliations:** ^1^School of Psychology, Shenzhen University, Shenzhen, China; ^2^Department of Psychology, University of Mannheim, Mannheim, Germany; ^3^Key Laboratory of Brain, Cognition and Education Sciences (South China Normal University), Ministry of Education, Guangzhou, China; ^4^School of Psychology, South China Normal University, Guangzhou, China; ^5^Center for Studies of Psychological Application, South China Normal University, Guangzhou, China; ^6^Guangdong Key Laboratory of Mental Health and Cognitive Science, South China Normal University, Guangzhou, China; ^7^Department of Psychology, Sun Yat-Sen University, Guangzhou, China; ^8^Shenzhen-Hong Kong Institute of Brain Science-Shenzhen Fundamental Research Institutions, Shenzhen, China

**Keywords:** fairness, framing effect, inequity aversion, advantage-seeking, computational model

## Abstract

Fairness plays a crucial role in children’s social life and has garnered considerable attention. However, previous research and theories primarily examined the development of children’s fairness behaviors in the conflict between self-interest motivation and fairness-complying motivation, neglecting the influence of advantage-seeking motivation. Moreover, despite the well-established role of gain/loss frame in human decision-making, it remains largely unclear whether the framing effect modulates fairness behaviors in children. It was hypothesized that children would exhibit advantage-seeking motivation resulting in more selfish behaviors in the loss context. To examine the hypothesis, we combined an adapted dictator game and computational modeling to investigate various motivations underlying fairness behaviors of children in both loss and gain contexts and to explore the developmental directions by contrasting children and adults. In addition, the current design enabled the dissociation between fairness knowledge and behaviors by asking participants to decide for themselves (the first-party role) or for others (the third-party role). This study recruited a total of 34 children (9–10 years, *M*_age_ = 9.82, *SD*_age_ = 0.38, 16 females) and 31 college students (*M*_age_ = 19.81, *SD*_age_ = 1.40, 17 females). The behavioral results indicated that children behaved more selfishly in first-party and more fairly in third-party than adults, without any significant framing effects. The computational results revealed that both children and adults exhibited aversion to advantageous and disadvantageous inequity in third-party. However, they showed distinct preferences for advantageous inequity in first-party, with advantage-seeking preferences among children and aversion to advantageous inequity among adults. These findings contribute to a deeper understanding of children’s social preferences and their developmental directions.

## 1 Introduction

Preferences for fairness represent a hallmark of human societies, playing a crucial role in interpersonal relationships and social cooperation (Brosnan and de Waal, 2014; Tomasello, 2016). It is also a key element of prosocial behavior that is positively linked to children’s classroom atmosphere (Layous et al., 2012) and academic performance (Caprara et al., 2000). Therefore, a better understanding of children’s fairness-related behaviors across contexts, especially their behavioral pattern and underlying psychological processes, is of theoretical and practical importance. The current study aimed to address the issue by combining an adapted dictator game (DG) and computational modeling to dissociate psychological subcomponents underlying children’s fairness decision-making across loss and gain frames. Moreover, this study explored the developmental direction of children’s fairness-related processes through a comparative analysis with adults.

Fairness development begins early in humans. Recent studies have found that even infants could express the preferences for fairness (Buyukozer Dawkins et al., 2019). In other words, infants could distinguish between fair and unfair allocations, and exhibit the expectation of fairness. The affiliative-preferences tasks were employed to examine the infants’ fairness preferences. In these tasks, one distributor divides resources equally between two recipients (fair-distributor event), and another distributor divides resources unequally (unfair-distributor event), infants are encouraged to choose between the two distributors by fixation time and reaching (Buyukozer Dawkins et al., 2019). Early as four-month-old infants already show an expectation for equal resource distribution, because they look longer at the unequal allocations (Buyukozer Dawkins et al., 2019) and could take into account distributors’ intentions (Geraci and Surian, 2023). Older infants and toddlers exhibit similar visual and reaching preferences, that is, they also consistently look longer at the unfair rather than the fair allocation and reach for the fair over the unfair distributor (Meristo et al., 2016; Enright et al., 2017; Bian et al., 2018; Franchin et al., 2019; Geraci et al., 2022). From age 3 and beyond, the fairness-related knowledge and behavior in children are usually examined with their choices in allocation tasks (Blake, 2018). Three years old children could express the knowledge of fairness by sharing resources equally in hypothetical allocation tasks or by allocating resources between two other people without sacrificing anything of their own (Olson and Spelke, 2008; Smith et al., 2013; Blake et al., 2014; Rizzo and Killen, 2016). However, their behavior usually falls short of their knowledge, showing the gap between knowledge and behavior, while the gap decreases as the age (Blake, 2018).

The majority of studies on children’s development in fairness behavior have been conducted within the framework of the seminal inequity aversion model (Fehr and Schmidt, 1999), such that these studies have presupposed that children are averse to the inequity between themselves and others. In other words, children dislike both disadvantageous inequity (others having more than self) and advantageous inequity (others having less than self), but to different extents. In accordance, children often exhibit preferences for the option with equal amounts of candies between themselves and others, instead of options with unequal candies favoring themselves or others. In particular, three-year-old children exhibit disadvantageous inequity aversion, while the emergence of advantageous inequity aversion tends to occur around the age of eight (Fehr et al., 2008; Blake and McAuliffe, 2011; McAuliffe et al., 2017; Li et al., 2022b). Several studies have indicated that children are capable of giving up the extra eraser to achieve equity between themselves and others even before the age of eight (Shaw and Olson, 2012; Antfolk et al., 2023).

The gap between fairness knowledge and behavior in children could be accounted for by the theory of behavioral control, which suggested that children require behavioral control to comply fairness in a conflict between self-interest and an equal outcome in advantageous inequity situation (McAuliffe et al., 2017; Blake, 2018). For instance, compared to younger children, children at age 8 exhibited advantageous inequity aversion, and they took longer to make allocation decisions, suggesting that children at age 8 started to control their behavior in line with social norms instead of making decisions based on selfish intuitions (Blake and McAuliffe, 2011). Additionally, the gap between fairness knowledge and behavior was negatively correlated with the scores of behavioral control skills (Blake et al., 2015b). Importantly, behavioral control improves considerably throughout childhood, becoming increasingly proactive, flexible and internalized (Munakata et al., 2012; McAuliffe et al., 2017). Therefore, the gap between knowledge and behavior decreases as behavioral control develops, while adults might internalize behavioral control in their fairness decision-making (Steinbeis et al., 2012).

In contrast to the predictions of the inequity aversion model and the theory of behavioral control, several lines of research have indicated that individuals prefer advantageous inequity over equity in some contexts. Firstly, 8-year-old children exhibit spiteful behavior during fairness-related decision-making, preferring options that benefit themselves while inflicting harm upon others (e.g., 1:0) over equal distribution (e.g., 1:1) (Fehr et al., 2008; Steinbeis and Singer, 2013). In the same vein, children may seek advantages over peers at the expense of personal cost. They may choose the option of 7:0 over 8:8 to maintain their superiority (Sheskin et al., 2014; Liu et al., 2017). With advancing age, spiteful behaviors tend to decrease, and aversion to inequity increases (Fehr et al., 2013; Steinbeis and Singer, 2013; McAuliffe et al., 2014; Sheskin et al., 2014). Nevertheless, even adults demonstrate both advantage-seeking and equal preferences, such that in response to unfair offers (e.g., 3:7) they sometimes compensate themselves to achieve an equal outcome (e.g., 7:7), but they may also choose to reverse the offer to seek an advantage (e.g., 7:3) (FeldmanHall et al., 2014). Second, both children and adults prefer to maintain existing unequal rankings at the expense of equity. They are reluctant to redistribute money between two anonymous others if this redistribution reverses initial rankings between others (Charness and Villeval, 2017; Xie et al., 2017; Li et al., 2022a). Third, many studies in the fields of psychology and cognitive neuroscience have indicated that individuals consider being better than others as rewards. For instance, comparing to those who are doing worse (i.e., downward comparison) often elevates positive emotions such as relief or schadenfreude and reduces anxiety (Amoroso and Walters, 1969; Gibbons, 1986; Jankowski and Takahashi, 2014). Consequently, downward comparison often enhances or protects subjective well-being (Suls et al., 2002). Likewise, downward comparison often evokes activation of the reward-related regions (e.g., ventral striatum) in a similar way as primary or monetary reward (Zink et al., 2008; Fliessbach et al., 2012; Bartra et al., 2013; Du et al., 2013; Kang et al., 2013; Zhang H et al., 2020). Taken together, advantage-seeking (i.e., being better than others) represents another fundamental motivation of human fairness decision-making. In the current study, we aimed to compare this hypothesis with the account of advantageous inequity aversion by employing computational models.

Moreover, fairness-related behaviors are modulated by loss-gain frame, such that individuals frequently show higher levels of fairness preferences in the loss frame than gain frame (Feng et al., 2021; Zhang et al., 2023). In particular, individuals propose more generous offers (Neumann et al., 2017; Yin et al., 2017; Thunström, 2019; Cochard et al., 2020), demand more equal offers from others, and are more likely to reject unfair offers (Camerer et al., 1993; Zhou and Wu, 2011; Guo et al., 2013) in the loss frame than in the gain frame. The impact of loss-gain context on human decision-making is thought to reflect the critical role of emotional or intuitive processes in human decision-making (Slovic et al., 2007). That is, the loss context, compared to the gain context, exacerbates intuitive reactions in response to the conflict between self-interest and prosocial preferences, regardless of whether those dominant responses are selfish or altruistic (Feng et al., 2021). Hence, as loss context weakens the behavioral control in children, they are likely to exhibit lower levels of fairness preferences. In contrast, adults are expected to exhibit higher levels of fairness preferences in the loss frame due to the internalized and intuitively activated behavioral control. Previous evidence has suggested that children are intuitively selfish (Chajes et al., 2022; Nava et al., 2023), while adults are intuitively cooperative and generous (Rand et al., 2012, 2014). Few studies have investigated the impact of loss-gain frame on children’s fairness behaviors, however, there is evidence showing that loss (vs. gain) frame increased children’s risky and dishonest behaviors (Markiewicz and Gawryluk, 2020; Rolison et al., 2022). In this regard, it was expected that loss (vs. gain) context would render children’s allocation less fair but adults’ allocation more fair.

Building on previous studies, the current study aimed to examine the impact of loss-gain frame on children’s knowledge and behaviors of fairness, to uncover the underlying cognitive mechanisms, and to unravel development directions by contrasting findings between children (about 10 years of age) and adults (about 20 years of age). Participants acted as dictators in a modified DG, in which they received an initial endowment of tokens and unilaterally made decisions, either for themselves or for others, to allocate some portion to an anonymous recipient. In each round, the relative gains and losses of dictators and recipients were manipulated by applying separate self and other multiplier ratios to convert tokens into payoffs for the dictators and the recipients, respectively. Leveraging the quantitative nature of the experimental design, we employed computational models to disentangle specific cognitive processes through which the framing effect influences children’s fairness behaviors/knowledge. Computational modeling aims to uncover cognitive processes driving observed behavioral patterns by employing mathematically precise equations (Hu et al., 2017; Gao et al., 2018; Husain and Roiser, 2018; Yu et al., 2018). Computational models reflect the instantiations of cognitive hypotheses, with the merit of allowing for precise predictions and quantitative test of competing hypotheses (Ahn et al., 2017; Wilson and Collins, 2019). In the current study, we investigated several hypotheses on the underlying cognitive processes of explicit fairness behaviors/knowledge as well as how loss-gain frame interacts with those processes to modulate fairness decision-making. For fairness behaviors, it was hypothesized that both children and adults were averse to disadvantageous inequity, while potentially divergent preferences for advantageous inequity: children might be indifferent to advantageous inequity and even exhibit advantage-seeking preferences; in contrast, adults would be averse to advantageous inequity (*Hypothesis 1*). Regarding knowledge of fairness, we expected no significant differences between children and adults (*Hypothesis 2*). Lastly, it was hypothesized that loss (vs. gain) frame would reduce inequity aversion and increase advantage-seeking motivation among children while increasing inequity aversion among adults (*Hypothesis 3*).

## 2 Materials and methods

### 2.1 Participants

A total of 34 children aged 9–10 years old (16 females, $M_{\mathrm{age}}=9.82,SD_{\mathrm{age}}=0.38$ ) and 31 college students (17 females, $M_{\mathrm{age}}=19.81,SD_{\mathrm{age}}=1.40$ ) participated in the current study. The sample size of current study was limited by the resources that are available (Lakens, 2022) and was comparable to the previous study on the similar topic (Morasse et al., 2022). The power sensitivity analysis was conducted with MorePower software (Campbell and Thompson, 2012). Using a 2 × 2 × 2 repeated-measures analysis of variance (ANOVA), effect size as small as η_p_^2^ = 0.114 (RM = 2 × 2, IM = 2, α = 0.05, 1 − β = 0.80) can be reliably detected given the current sample size (Bakeman, 2005). The child participants were recruited from a primary school in Chengdu, China, and the adult participants were recruited from South China Normal University, Guangzhou, China. The Ethics Committee of South China Normal University approved the experimental design and participant recruitment. All participants were screened to ensure that they did not have any current or past neurological or psychiatric disorders, and provided informed consent before experiment.

### 2.2 Design and material

In the current study, a mixed 2 × 2 × 2 design was employed with Role (first-party or third-party) and Frame (gain or loss) as within-subjects factors and Group (children or adults) as a between-subjects factor. Moreover, we employed a modified version of DG with expanded choice space to better explore the effects of both disadvantageous and advantageous inequity aversion (Sáez et al., 2015).

**Figure 1:**
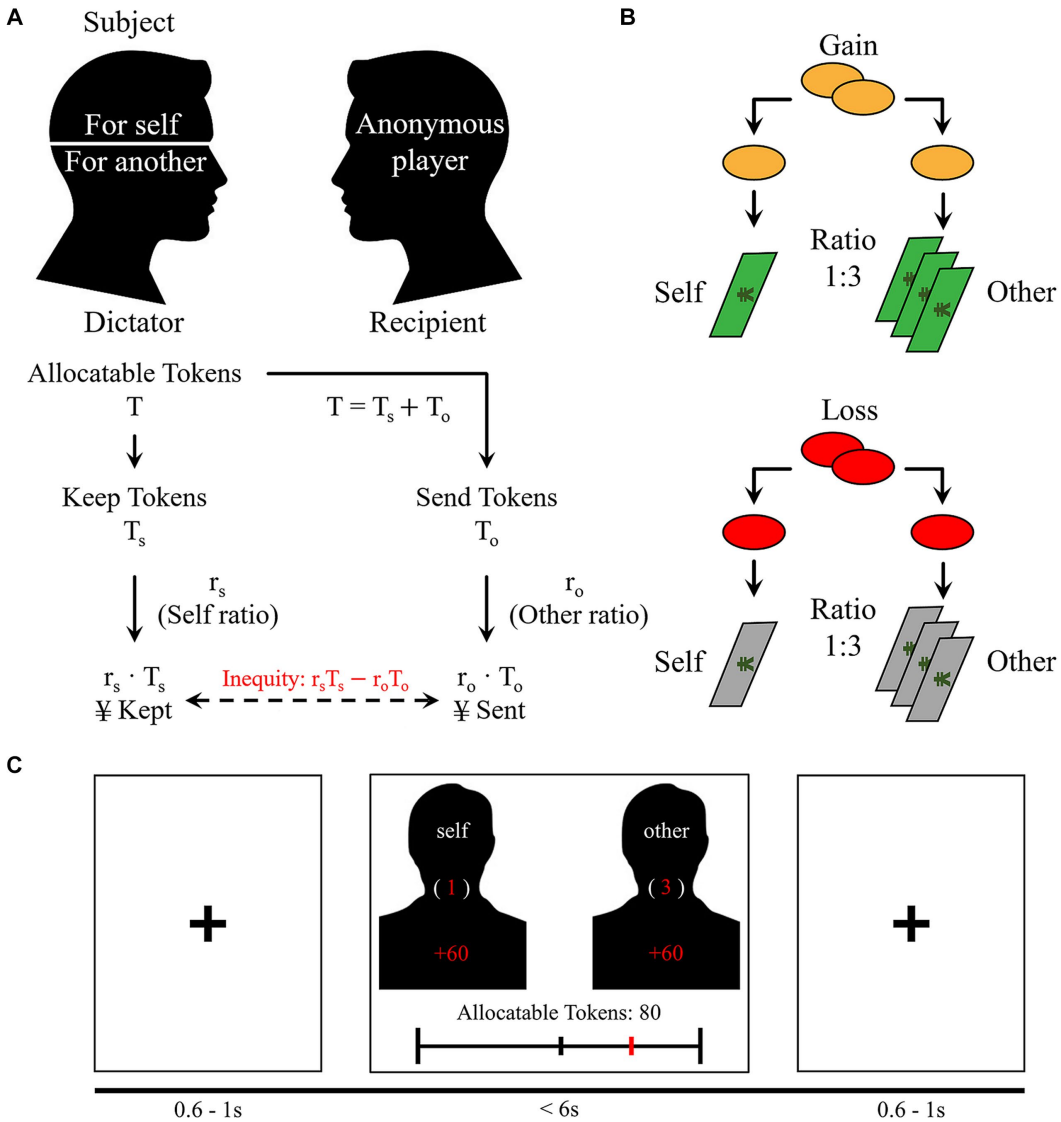
Experimental paradigm. **(A)** The dictator game. Participants acted as dictators in the role of first-party or third-party. Inequity was defined as the difference between the payoffs of both players. **(B)** Frames of the game. In the gain frame, allocatable tokens were exchanged for cents, while in the loss frame, tokens were exchanged to a loss in cents. Tokens were multiplied by a ratio (3:1, 2:1, 1:1, 1:2, and 1:3) when exchanged for cents. **(C)** The timeline of the DG task. Each round started with a jitter of 0.6 s ~ 1.0 s, followed by a decision phase, in which participant made their decisions within 6 s [The figure was inspired by (Sáez et al., 2015)].

As in the standard DG, participants received a sum of tokens and were allowed to allocate some tokens (T_o_) to an anonymous recipient while keeping the remaining tokens (T_s_) for themselves (Camerer, 2011). The value of each token to the dictator (r_s_) and the recipient (r_o_) varied according to exchange ratios (Figure 1A). Taking a ratio of 1:3 as an example, 1 token was worth 1 cent to the participant, while 1 token was worth 3 cents to the recipient (Figure 1B, gain frame). Five exchange ratios were included in the experiment (3:1, 2:1, 1:1, 1:2, and 1:3), and the amounts of allocable tokens varied in each round.

Participants played the game in different roles (first-party versus third-party) and in different frames (gain versus loss), resulting in four conditions in a sequence determined by a Latin Square design. In the role of first-party, participants made decisions for themselves, so their decisions directly impacted the amount of cents they would earn or lose for themselves and anonymous recipients. In the role of third-party, they made decisions for another anonymous dictator and allocated tokens between the dictator and recipients (Figure 1A). Therefore, their choices did not affect their own interests but those of an anonymous dictator and recipients. Participants allocated gains in the gain frame while they allocated losses in the loss frame (Figure 1B).

The four conditions were presented to participants in different blocks, with the order of the conditions counterbalanced across participants. Before each block, participants were informed of their current role and frame. Each block consisted of 20 rounds. Each round began with a jitter between 0.6 s and 1 s. Afterwards, the amounts of allocable tokens as well as the exchange ratio were presented, based on which participants made their decisions by moving the mouse to the desired amount (Figure 1C). Participants were instructed to make their decisions within 6 s.

### 2.3 Statistical analysis of explicit behaviors

To investigate the effects of self-interest and equity on participants’ behaviors, the extents to which participants’ choices deviate from selfish and equal rules were, respectively, computed. Noteworthy, to control for the confounding of varying numbers of allocable tokens in each round, participants’ decisions in each round were computed as the number of tokens kept by participants (T_s_) divided by allocable tokens (T) (Jiang et al., 2015). In the same vein, the choices predicted by selfish and equal rules were also expressed as ratios to the number of allocable tokens. Those behavioral measures were then examined using repeated-measures analyses of variance (ANOVAs), with Frame (gain versus loss) and Role (first-party versus third-party) as within-subjects factors, and Group (children versus adults) as a between-subjects factor.

#### 2.3.1 Selfish deviation

Selfish deviation was defined as the absolute difference between the choice predicted by selfish rule and participants’ choice. The perfect selfish choice is guided by the perfect selfish utility hypothesis, which suggests that participants aimed to maximize their personal interests (r_s_T_s_) regardless of inequity. A perfect selfish participant would keep all the allocable tokens in gain frame, while sending all the tokens in loss frame. Therefore, the perfect selfish option would be to keep 100% in gain condition and 0% in loss condition. Selfish deviation was calculated by the absolute difference between 100% and participants’ choices in gain frame, and by the absolute difference between 0% and participants’ choices in loss frame. For instance, participants received 3 tokens and kept 1 token, in the gain frame, selfish deviation was the absolute difference between 100 and 33% (participant’s choice, T_s_ = 1; T = 3), that is 77%; in the loss frame, selfish deviation was the absolute difference between 0 and 33% (participant’s choice) in loss frame, that is 33%. The selfish deviation was independent of the exchange ratios.

#### 2.3.2 Equal deviation

Equal deviation was defined as the absolute difference between the choice predicted by equal rules and participants’ choice. The perfect equal behavior was driven by the inequity aversion, which motivated participants to minimize the inequity (r_s_T_s_ − r_o_T_o_) regardless of frame. A perfect equal participant would ensure the inequity was 0 (r_s_T_s_ = r_o_T_o_), therefore, the amount of tokens kept would satisfy the formula: T_s_ = r_o_T_o_/r_s_. The perfect equal choices could be computed as (r_o_T_o_/r_s_)/T. For instance, with the exchange ratio of 1:3, the perfect equal choice would be keeping 75% (r_o_T_o_/r_s_ = 3T_o_, T = 4T_o_). Therefore, if participants received 3 tokens and kept 1 token, the equal deviation would be the absolute difference between 75 and 33% (participant’s choice, T_s_ = 1; T = 3), that is 42%.

### 2.4 Computational modeling

#### 2.4.1 Model construction

Assuming that both children and adult participants’ decisions were modulated by self-interest and concerns for inequity, we employed several alternative economic models to uncover the cognitive processes underlying the effects of different contexts on distributive behaviors in adults and children, respectively. Firstly, two models were designed to examine whether participants only focused on one type of inequity.

*Advantageous inequity aversion model* (*M1*, Equation 1) assumes that participants’ decisions are only modulated by concerns for advantageous inequity, which defines the subjective value function as:

(1) $$U=Ms-\alpha_{k}*\max\left(Ms-Mo,0\right)$$

Where *U* represents the utility of each option. *Ms* and *Mo* refer to self-payoff and other-payoff, respectively. The monetary payoffs for self and the other are calculated as *Ms* = r_s_T_s_ and *Mo* = r_o_T_o_. The parameters *α_k_* (−∞ < *α_k_* < +∞) quantify subjective aversion to advantageous inequity. The parameters vary across conditions and participants, such that larger parameter values indicate a higher level of concern of advantageous inequity. The aversion to inequity is captured by the parameters *α_k_*, with k = {1, 2, 3, 4} respectively corresponding to the conditions of first-party in the gain context, first-party in the loss context, third-party in the gain context and third-party in the loss context. A larger *α_k_* (*α_k_* > 0) indicates participants are more averse to advantageous inequity, while a smaller *α_k_* (*α_k_* < 0) indicates participants are more prefer advantageous inequity.

*Disadvantageous inequity aversion model* (*M2*, Equation 2), on the other hand, assumes the utility would only focus on disadvantageous inequity, which defines the subjective value function as:

(2) $$U=Ms-\beta_{k}*\max\left(Mo-Ms,0\right)$$

The parameters *β_k_* (−∞ < *β_k_* < +∞) quantify subjective aversion to disadvantageous inequity.

Secondly, two models were designed to examine whether participants could differentiate between two types of inequity.

*General inequity aversion model* (*M3*, Equation 3) assumes participants’ utility would not dissociate the weights on advantageous and disadvantageous inequity aversion separately, which indicates the participants’ aversion to inequity in general (Gao et al., 2018), which defines the subjective value function as:

(3) $$U=Ms-\omega_{k}*\left|Ms-Mo\right|$$

Where parameters *ω_k_* (−∞ < *ω_k_* < +∞) characterize the weight on general inequity aversion.

*Fehr-Schmidt inequity aversion model (M4*, Equation 4*)* assumes that the utility of each option varies with participants’ self-payoff and their aversion to inequity, and the weights on advantageous and disadvantageous inequity aversion are dissociated in decision-making (Sáez et al., 2015; Gao et al., 2018; Morasse et al., 2022), which could be computed as the following formula:

(4) $$U=Ms-\alpha_{k}*\max\left(Ms-Mo,0\right)-\beta_{k}*\max\left(Mo-Ms,0\right)$$

Accordingly, the parameters *α_k_* (−∞ < *α_k_* < +∞) and *β_k_* (−∞ < *β_k_* < +∞) respectively quantify subjective aversion to advantageous and disadvantageous inequity. Moreover, we construct another model *(M5)* with parameters constrained to values higher than zero (*α_k_* > 0, *β_k_* > 0).

Based on the utility of each option, the probability of choice is determined by the softmax function (Equation 5):

(5) $$P\left(Ms,Mo\right)=\frac{e^{\lambda \cdot U}\left(Ms,Mo\right)}{\sum_{j\in J}e^{\lambda \cdot U}\left(Ms^{j},Mo^{j}\right)}$$

Where *P* denotes the probability of choosing specific allocation (*Ms*, *Mo*) in each trial. The *λ* (*λ* > 0) refers to inverse softmax temperature parameter capturing the stochasticity of participants’ decisions, that is, lower *λ* corresponds to more diffuse and variable choices.

#### 2.4.2 Parameter estimation

Parameter estimation was conducted with hierarchical Bayesian Analysis (HBA), which enables a more stable and precise estimation compared to maximum likelihood estimation (Ahn et al., 2013). The HBA was implemented with the ‘Rstan’ package in R environment, which adopts a Markov Chain Monte Carlo (MCMC) sampling method to execute fully-Bayesian inference and get the posterior distributions (Carpenter et al., 2017; Johnson et al., 2022). Prior to sampling, the prior distributions for group and individual parameters were needed to define separately, and it was proposed that individual level parameters were drawn from group level normal distribution $N\left(\mu_{0},\sigma_{0}\right)$ where $\mu_{0}\;\mathrm{and}\;\sigma_{0}$ refer to group-level mean and standard deviation, in other words, the hyper parameters of individual-level parameters (Ahn et al., 2017). Weakly informative priors were adopted for the priors of the group-level normal means and standard deviations: *μ_0_* ~ Normal (0, 1) and *σ_0_* ~ half-Cauchy (0, 2). This was to minimize the impact of priors on the posterior distributions. Model and priors were defined in a stan file which was then compiled in R environment. Each model was fitted with 3 MCMC chains each of which contains 2000 iterations after 2000 iterations for warmup, yielding a total of 6,000 saved simulation draws. The convergence of MCMC chains was adequate, according to the trace plot and the Gelman-Rubin R-hat statistics (R-hat values of all parameters are close to 1.0, smaller than 1.01) (Brooks and Gelman, 1998).

#### 2.4.3 Model comparison

For model comparison, the leave-one-out information criterion (LOOIC) and widely applicable information criterion (WAIC) were calculated as the criteria for model comparison using ‘loo’ package for R, as recommended in recent studies (Vehtari et al., 2017). Both LOOIC and WAIC represent the estimation of out-of-sample pointwise predictive accuracy using the posterior simulations in a totally Bayesian way. LOOIC uses leave-one-out cross-validation and WAIC is based on the series expansion of leave-one-out cross-validation (Vehtari et al., 2017). By convention, the lower LOOIC or WAIC indicates better prediction accuracy of candidate models, thus the model with the lowest WAIC and LOOIC is the winning one. In general, a 10-point difference of LOOIC or WAIC between two models can demonstrate the superiority (Burnham and Anderson, 2004). LOOIC and WAIC of all candidate models are computed with the “loo” package in R.

Based on the difference in LOOIC/WAIC value between each candidate model and the winning model, it is also possible to obtain LOOIC/WAIC weights. These weights indicate the probability of being the best model for each model, given the data and the set of candidate models (Hoeting et al., 1999).

#### 2.4.4 Model validation

In light of the analysis scheme recommended in recent tutorial papers (Zhang L et al., 2020), a posterior predictive check (PPC) was implemented to ensure the model fits data. Using the parameters’ joint posterior distribution obtained from the winning model, new synthetic choice data was generated individually and then correlated with the actual behavioral results across participants and trials, respectively. Moreover, the simulated data was forwarded to the statistical analyses performed for the actual data, to recover the same effect on behavior observation.

## 3 Results

### 3.1 Explicit behaviors

The optimal choices predicted by selfish and equal rules across different conditions are shown in Table 1.

**Table 1:** Optimal choices (i.e., percentage of tokens kept) predicted by selfish and equal rules.

Self: Other ratio	Gain		Loss	
	Selfish	Equal	Selfish	Equal
1:3	100%	75%	0%	75%
1:2	100%	67%	0%	67%
1:1	100%	50%	0%	50%
2:1	100%	33%	0%	33%
3:1	100%	25%	0%	25%

#### 3.1.1 Modulations of roles on selfish deviations

**Figure 2:**
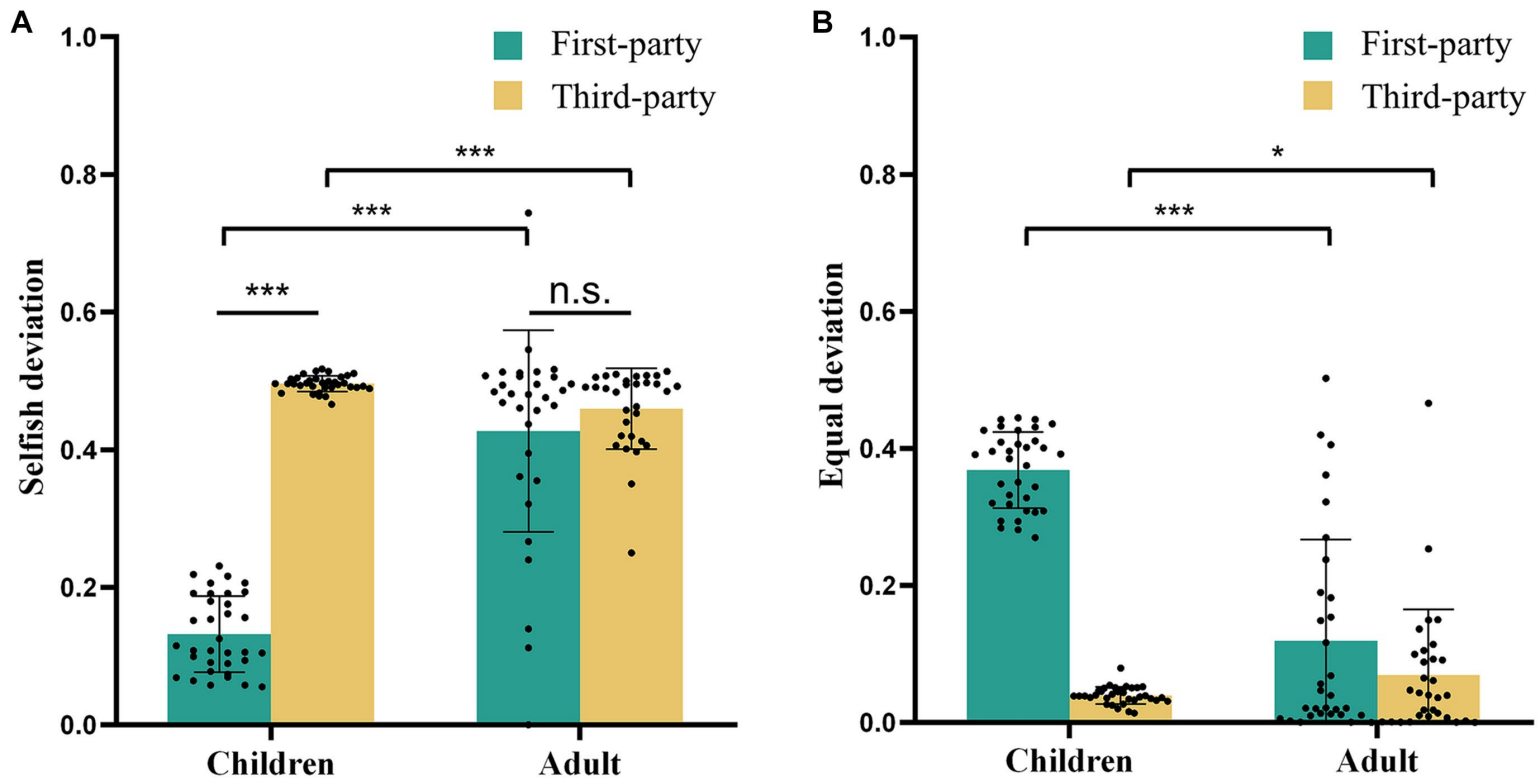
Behavioral results. **(A)** The selfish deviations. For children, selfish deviations were higher in the role of first-party than third-party, while there was no significant difference between roles for adults. Moreover, children showed lower selfish deviations than adults in first-party role, but higher deviations than adults in third-party role. **(B)** The equal deviations. Equal deviations were higher for children than adults in first-party role, but lower for children than adults in third-party role. ^*^p < 0.05, ^***^p < 0.001. n.s., not significant.

The interaction of Role and Group was significant [*F*_(1, 63)_ = 180.507, *p* < 0.0005, *η*_p_^2^ = 0.741]. Post-hoc comparisons indicated that children showed lower selfish deviations than adults in first-party role (*mean difference* ± *s.e.* = −0.297 ± 0.027, *p* < 0.0005, Figure 2A), while children showed higher selfish deviations than adults in third-party role (*mean difference* ± *s.e.* = 0.036 ± 0.010, *p* = 0.0011, Figure 2A). These findings suggested that children acted more selfishly than adults in first-party role, but less selfishly in third-party role.

Moreover, for children, selfish deviations were higher in the role of third-party than first-party (*mean difference* ± *s.e.* = 0.364 ± 0.017, *p* < 0.0005, Figure 2A), while there was no significant difference between roles for adults (*mean difference* ± *s.e.* = 0.031 ± 0.018, *p* = 0.0918, Figure 2A). These results suggested that children showed a gap between knowledge and behavior, while adults did not exhibit this gap.

#### 3.1.2 Modulations of roles on equal deviations

A significant interaction effect of Role and Group was observed [*F*_(1, 63)_ = 179.292, *p* < 0.0005, *η*_p_^2^ = 0.740]. Post-hoc comparisons indicated that equal deviations were higher for children than adults in first-party role (*mean difference* ± *s.e.* = 0.245 ± 0.028, *p* < 0.0005, Figure 2B), but lower for children than adults in third-party role (*mean difference* ± *s.e.* = −0.036 ± 0.017, *p* = 0.0371, Figure 2B). These results suggested that while children showed more concerns for equity in the third-party role, they showed less concern for equity when their personal interests were involved.

Together, children and adults exhibited distinct fairness-related preferences in first- and third-party roles. It is noteworthy that the results indicate no significant effect of the Frame on participants’ decision-making. The full list of effects in the ANOVA was reported in the Supplementary Tables S1, S2.

### 3.2 Model-based analyses

#### 3.2.1 Model comparison

**Figure 3:**
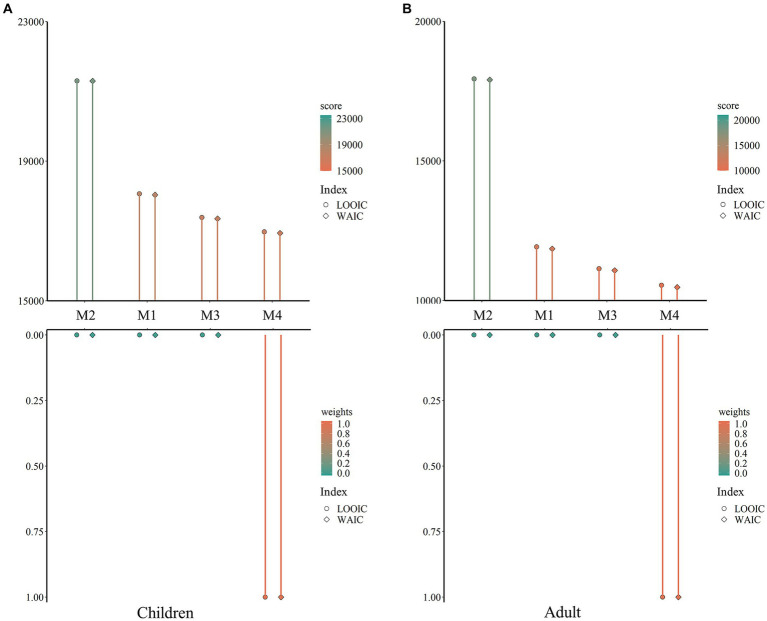
Results of model comparison. **(A)** The scores and weights of LOOIC and WAIC across models applied to child participants. Dots and triangles indicate summed LOOIC and WAIC scores for each model from largest (green) to smallest (orange) respectively, while dots and triangles indicate summed LOOIC and WAIC weights for each model from largest (orange) to smallest (green) respectively. **(B)** The scores and weights of LOOIC and WAIC across models applied to adult participants. These results indicate that the Fehr-Schmidt inequity aversion model (Model 1) best captured choices of children and adult participants compared against the other three models.

Four computational models were constructed and compared to elucidate the cognitive processes underlying children and adult participants’ decision-making. The results indicated that, regardless of participants’ group, *Fehr-Schmidt inequity aversion model (M4)* exhibited the lowest scores and the highest weights of both LOOIC and WAIC, which indicated the superiority of M4 compared to the other three models (Figures 3A,B). Remarkably, we constructed the same model (M5) as M4, with parameters constrained to values higher than zero. Children’s results revealed that M5 exhibited higher LOOIC and WAIC scores than M4.

#### 3.2.2 Model validation

To further validate the winning model, choices from actual participants and model predictions (the simulated behaviors generated from winning model based on the estimated participants’ parameters) were compared. Strong correlations were observed between model predictions and actual behaviors for both age groups. For children, significant correlations were found between model predictions and actual behaviors across participants (first-party role in the gain frame, *n* = 34, *r* = 0.938, *p* < 0.001; third-party role in the gain frame, *n* = 34, *r* = 0.911, *p* < 0.001; first-party role in the loss frame, *n* = 34, *r* = 0.734, *p* < 0.001; third-party role in the loss frame, *n* = 34, *r* = 0.747, *p* < 0.001; see Supplementary Figures S2–S5) and across trials (first-party role in the gain frame, *n* = 20, *r* = 0.996, *p* < 0.001; third-party role in the gain frame, *n* = 20, *r* = 0.820, *p* < 0.001; first-party role in the loss frame, *n* = 20, *r* = 0.998, *p* < 0.001; third-party role in the loss frame, *n* = 20, *r* = 0.999, *p* < 0.001; see Supplementary Figures S2–S5). Likewise, for adults, the significant correlations were found across participants (first-party role in the gain frame, *n* = 31, *r* = 0.989, *p* < 0.001; third-party role in the gain frame, *n* = 31, *r* = 0.995, *p* < 0.001; first-party role in the loss frame, *n* = 31, *r* = 0.931, *p* < 0.001; third-party role in the loss frame, *n* = 31, *r* = 0.970, *p* < 0.001; see Supplementary Figures S6–S9) and trials (first-party role in the gain frame, *n* = 20, *r* = 0.997, *p* < 0.001; third-party role in the gain frame, *n* = 20, *r* = 0.998, *p* < 0.001; first-party role in the loss frame, *n* = 20, *r* = 0.994, *p* < 0.001; third-party role in the loss frame, *n* = 20, *r* = 0.996, *p* < 0.001; see Supplementary Figures S6–S9).

**Figure 4:**
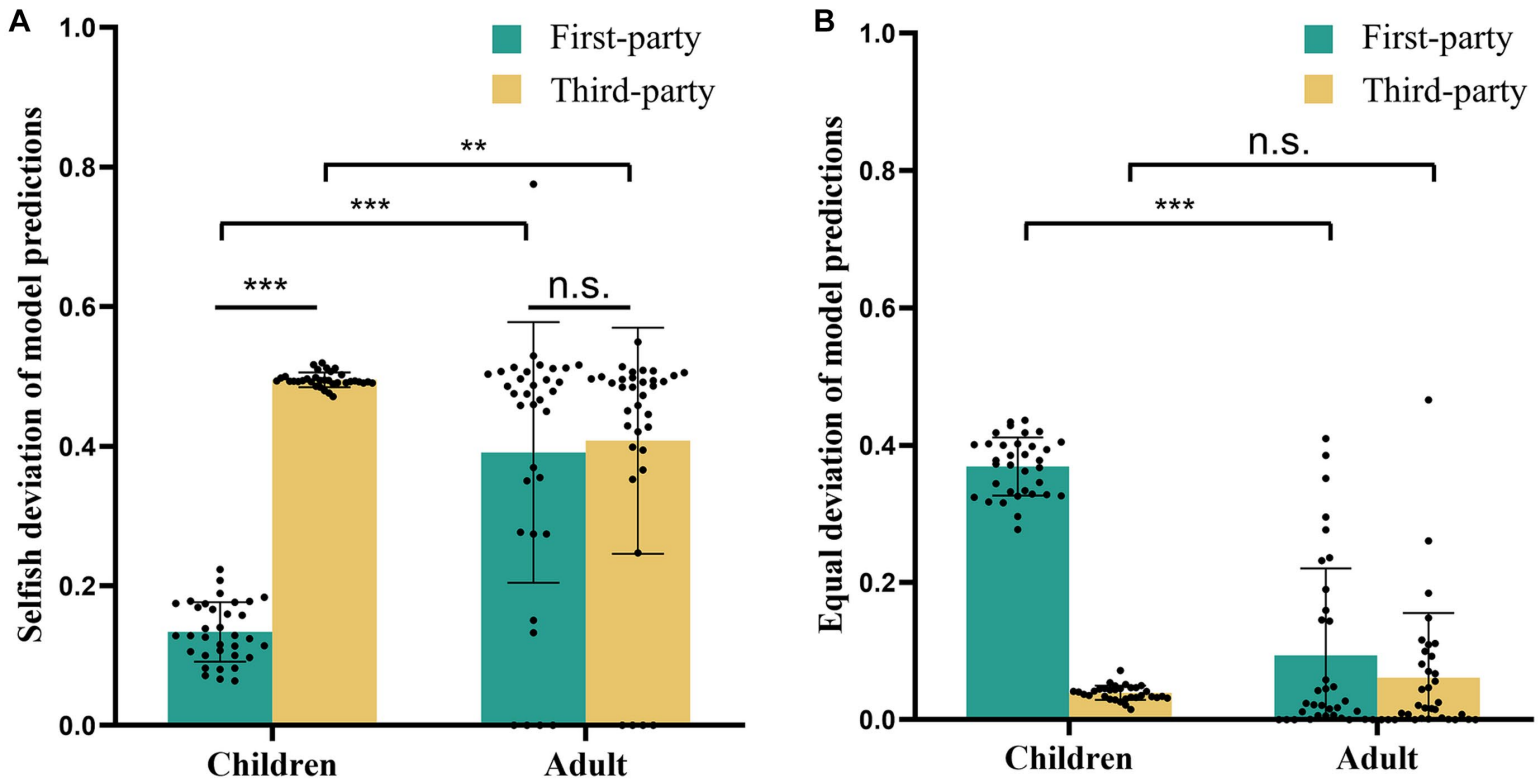
Behavioral results of simulated data. **(A)** The average of selfish deviation. For children, selfish deviations were higher in the role of first-party than third-party, while there was no significant difference between roles for adults. Moreover, children showed lower selfish deviations than adults in first-party role, but higher deviations than adults in third-party. **(B)** The average of equal deviation. Equal deviations were higher for children than adults in first-party role, but exhibited no significance between children than adults in third-party role. ^**^p < 0.005, ^***^p < 0.001. n.s., not significant.

Finally, simulated behaviors performed a great behavioral recovery, consisted with the findings from real behavioral analyses:

(1) For selfish deviations (Figure 4A), the interaction of Role and Group remained significant [*F*_(1, 63)_ = 172.254, *p* < 0.001, *η*_p_^2^ = 0.732]. Post-hoc comparisons indicated that children showed lower selfish deviations than adults in first-party role (*mean difference* ± *s.e.* = −0.291 ± 0.027, *p* < 0.0005), while children showed higher selfish deviations than adults in third-party role (*mean difference* ± *s.e.* = 0.033 ± 0.011, *p* = 0.0028). Moreover, for children, selfish deviations were higher in the role of third-party than first-party (*mean difference* ± *s.e.* = 0.357 ± 0.017, *p* < 0.0005), while there was no significant difference between roles for adults (*mean difference* ± *s.e.* = 0.033 ± 0.018, *p* = 0.0709).

(2) For equal deviations (Figure 4B), the significant interaction effect of Role and Group was observed [*F*_(1, 63)_ = 240.450, *p* < 0.001, *η*_p_^2^ = 0.792]. Post-hoc comparisons indicated that equal deviations were higher for children than adults in first-party role (*mean difference* ± *s.e.* = 0.253 ± 0.024, *p* < 0.0005), while there was no significant difference between children and adults in third-party role (*mean difference* ± *s.e.* = −0.030 ± 0.017, *p* = 0.0790). Although the latter result is not statistically significant and not precisely align with the actual result, the simulated data consistently demonstrated the correct trend. Overall, these results demonstrated that our model worked well and captured key psychological components of participants’ decision-making.

#### 3.2.3 Differences in fairness-related preferences between children and adults

In subsequent analyses, group-level parameters of the winning model, including advantageous and disadvantageous inequity aversion parameters (*α* and *β*, respectively), were estimated through hierarchical Bayesian Analysis. Meaningful differences were determined if 97.5% highest density interval (HDI) of the posterior was above or below 0 and 85–97.4% HDI of the posterior above or below 0 was interpreted as providing more limited evidence for an effect (Brown et al., 2022). Firstly, the difference between estimated parameters and 0 was examined.

**Figure 5:**
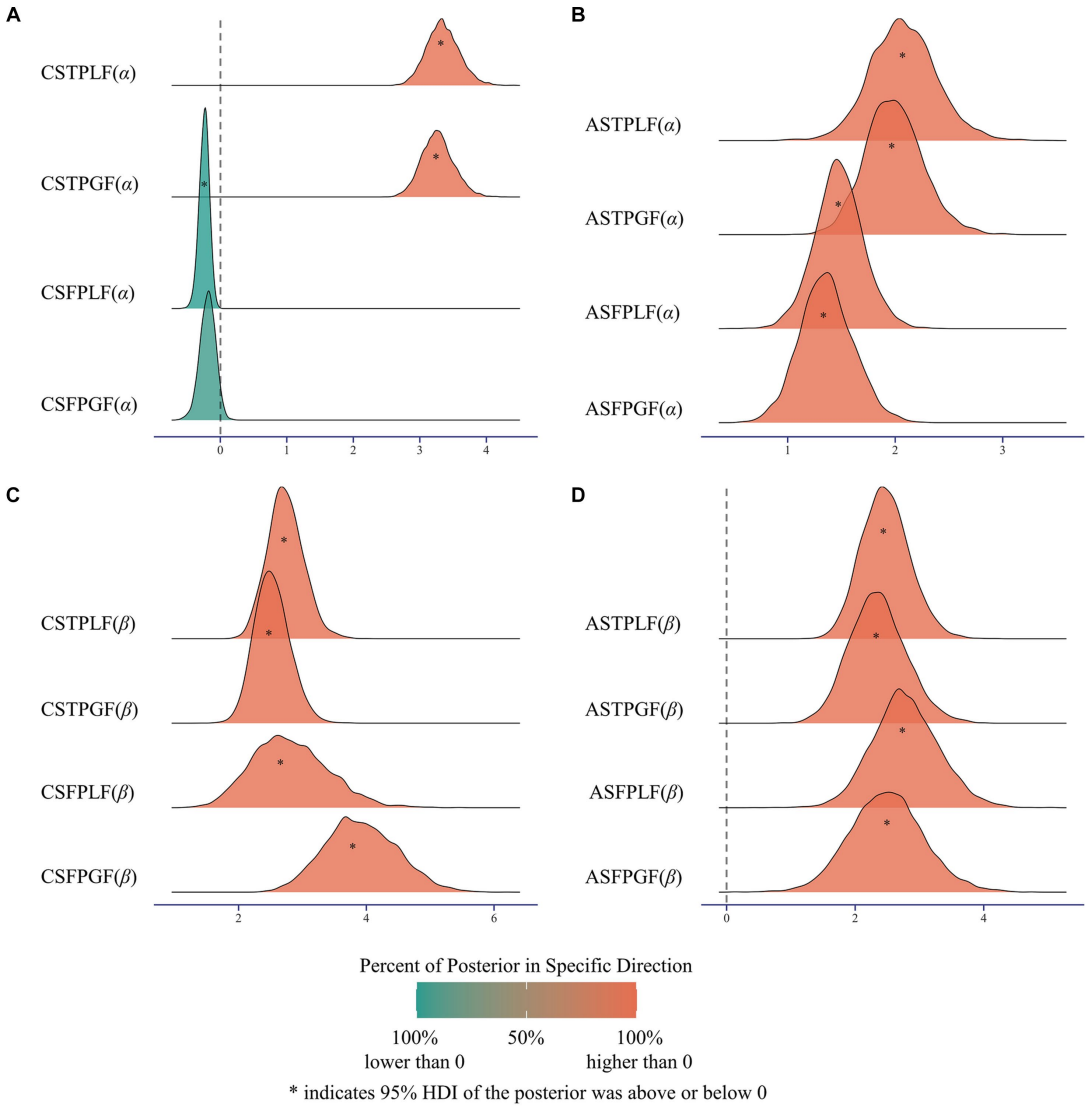
Results of the difference between group-level parameters and 0. **(A)** Children’s advantageous aversion parameters (*α*) were lower than 0 in the loss frame but not significant different in the gain frame for the role of first-party. Children’s advantageous aversion parameters (*α*) were higher than 0 in the gain frame and loss frame for third-party role. **(B)** adults’ advantageous aversion parameters (*α*) were higher than 0 in first-party and gain frame, first-party and loss frame, third-party and gain frame and third-party and loss frame. **(C)** Children’s disadvantageous aversion parameters (*β*) were higher than 0 in first-party and gain frame, first-party and loss frame, third-party and gain frame and third-party and loss frame. **(D)** Adults’ disadvantageous aversion parameters (*β*) were higher than 0 in first-party and gain frame, first-party and loss frame, third-party and gain frame and third-party and loss frame. FP: first-party; TP: third-party; CS, children; AS, adults; GF, gain frame; LF, loss frame.

When children acting as first-party, parameters of aversion to advantageous inequity (*α*) were lower than 0 in the loss frame (mean = −0.241, 95% HDI: [−0.387, −0.103], Figure 5A), and marginally lower than 0 in the gain frame (mean = −0.190, 95% HDI: [−0.407, 0.046], Figure 5A). When children acting as third-party, the parameters of aversion to disadvantageous inequity were higher than 0 in both gain frame (mean = 3.268, 95% HDI: [2.805, 3.751], Figure 5A) and loss frame (mean = 3.348, 95% HDI: [2.873, 3.835], Figure 5A). For adults, parameters of aversion to advantageous inequity were higher than 0 across four conditions: first-party in the gain frame (mean = 1.361, 95% HDI: [0.869, 1.846], Figure 5B), first-party in the loss frame (mean = 1.478, 95% HDI: [1.057, 1.967], Figure 5B), third-party in the gain frame (mean = 1.992, 95% HDI: [1.468, 2.526], Figure 5B) and third-party in the loss frame (mean = 2.077, 95% HDI: [1.471, 2.688], Figure 5B). These results indicated that children did not exhibit aversion but rather a preference for advantageous inequity in the first-party, while adults exhibited aversion for advantageous inequity across conditions. Moreover, children and adults exhibited aversion to advantageous inequity in third-party.

When children acting as first-party, parameters of aversion to disadvantageous inequity (*β*) were higher than 0 in both gain frame (mean = 3.919, 95% HDI: [2.884, 5.035], Figure 5C) and loss frame (mean = 2.809, 95% HDI: [1.703, 3.959], Figure 5C). When children acting as third-party, parameters of aversion to disadvantageous inequity (*β*) were higher than 0 in both gain frame (mean = 2.525, 95% HDI: [2.022, 3.081], Figure 5C) and loss frame (mean = 2.735, 95% HDI: [2.197, 3.263], Figure 5C). For adults, parameters of aversion to disadvantageous inequity were higher than 0 across four conditions: first-party in the gain frame (mean = 2.489, 95% HDI: [1.317, 3.595], Figure 5D), first-party in the loss frame (mean = 2.784, 95% HDI: [1.802, 3.823], Figure 5D), third-party in the gain frame (mean = 2.353, 95% HDI: [1.524, 3.260], Figure 5D) and third-party in the loss frame (mean = 2.476, 95% HDI: [1.788, 3.242], Figure 5D). Therefore, both children and adults exhibited aversion to disadvantageous inequity. Taken together, children and adults shared similar behavioral preferences in third-party, while distinct behavioral preferences in first-party.

**Figure 6:**
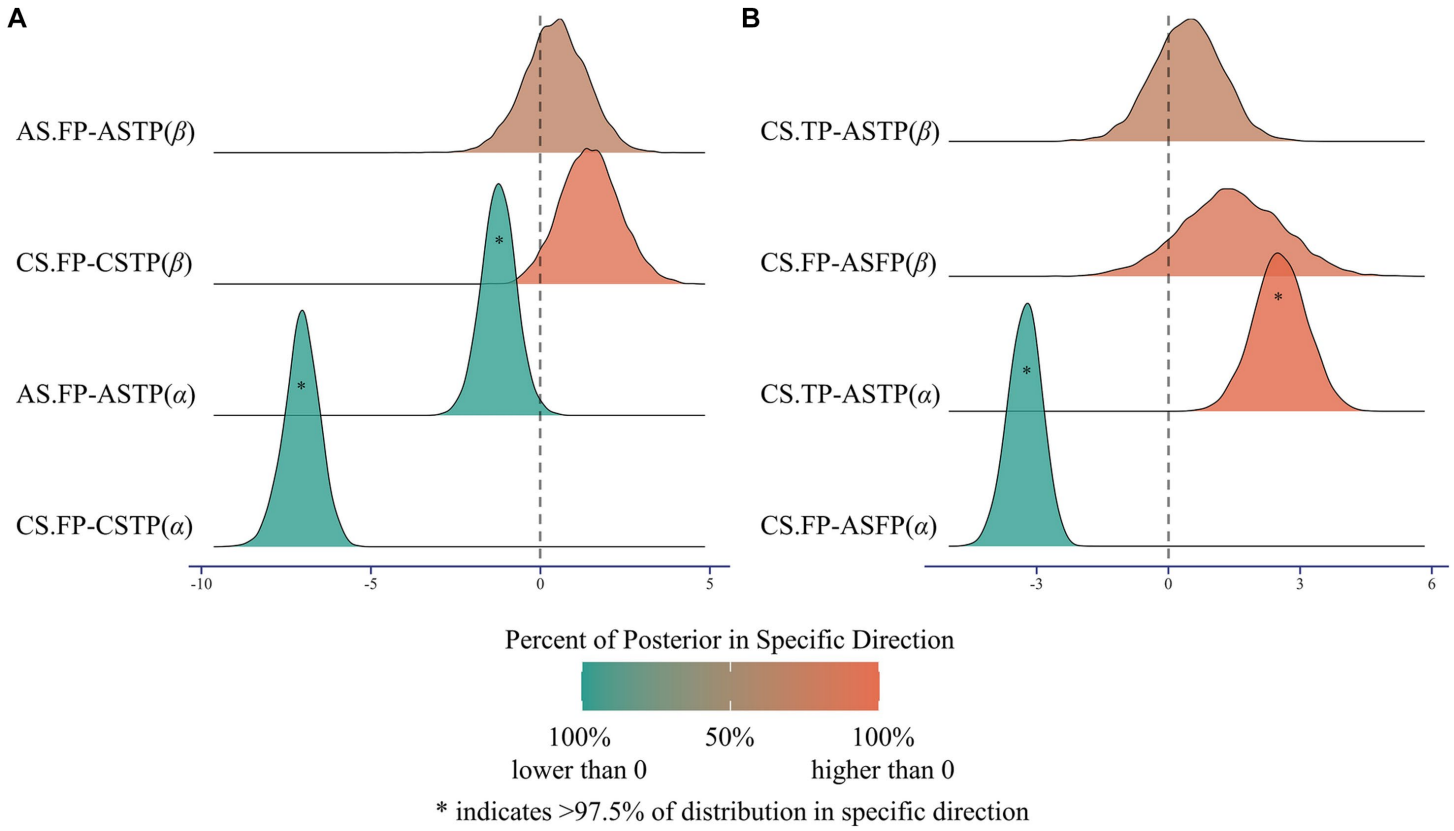
Results of the difference between group-level parameters. **(A)** The effect of Role. Children’s advantageous aversion parameters (*α*) were lower in the role of first-party than third-party, while disadvantageous aversion parameters (*β*) were not significant in the role of first-party than third-party. In adults’ condition, advantageous aversion parameters (*α*) were lower in the role of first-party than third-party, while disadvantageous aversion parameters (*β*) were not significant in the role of first-party than third-party. **(B)** The effect of Group. Advantageous aversion parameters (*α*) were lower for children than adults in first-party role, while were higher for children than adults in third-party role. Moreover, there were no significance in disadvantageous aversion parameters (*β*) for children and adults in first-party role and third-party role. The shading and asterisks indicating the percentage of samples from the posterior greater than or less than 0. FP, first-party; TP, third-party; CS, children; AS, adults.

Secondly, the difference between the estimated parameters across conditions was examined. For children, parameters of aversion to advantageous inequity were lower in the role of first-party than third-party (mean = −7.046, 95% HDI: [−8.104, −6.024], Figure 6A), while parameters of aversion to disadvantageous inequity showed no significant difference between roles (mean = 1.468, 95% HDI: [−0.271, 3.129], Figure 6A). For adults, parameters of aversion to advantageous inequity were lower in the role of first-party than third-party (mean = −1.231, 95% HDI: [−2.216, −0.190], Figure 6A), while parameters of aversion to disadvantageous inequity showed no significant difference between roles (mean = 0.445, 95% HDI: [−1.315, 2.242], Figure 6A).

Moreover, parameters of aversion to advantageous inequity were lower in children than adults as first-party role (mean = −3.269, 95% HDI: [−3.993, −2.506], Figure 6B), but higher in children than adults as third-party role (mean = 2.546, 95% HDI: [1.272, 3.630], Figure 6B). In contrast, there was no significant difference between children and adults for parameters of aversion to disadvantageous inequity either in first-party role (mean = 1.454, 95% HDI: [−0.713, 3.706], Figure 6B) or third-party role (mean = 0.431, 95% HDI: [−1.219, 1.827], Figure 6B). These results suggested that differences in fairness behaviors between children and adults primarily stem from alterations in aversions/preferences of advantageous inequity, but not that of disadvantageous inequity.

Lastly, parameters of aversion to advantageous or disadvantageous inequity did not differ between gain/loss frames, as illustrated in Supplementary Figure S1.

## 4 Discussion

Combining an adapted Dictator Game (DG) task that manipulates the loss/gain frame and first-party/third-party role, and computational modeling that enables us to disentangle cognitive processes, the current study examined preferences and motivations underlying children’s fairness-related knowledge and behavior as well as their developmental directions. Our results revealed that both children and adults acquired fairness knowledge, such that their decisions for others (i.e., as third-party) were well accounted for by equity norms, with children exhibiting higher preferences for equity than adults. However, children and adults exhibited distinct fairness behaviors when acting as first-party. On one hand, adults exhibited aversion to both advantageous and disadvantageous inequity, mirroring their fairness knowledge. On the other hand, children were also averse to disadvantageous inequity, but they exhibited advantage-seeking preferences instead of aversion to advantageous inequity according to computational modeling results. These findings did not only corroborate gaps in fairness-related knowledge and behaviors among children, but also provide novel evidence on advantage-seeking preferences pertaining to resource distribution. Lastly, the current study did not reveal significant effects of loss/gain frame on fairness-related knowledge or behaviors among either children or adults.

When deciding for others, children and adults showed aversion to both advantageous and disadvantageous inequity, demonstrating that 10-year-old children have acquired the principle of fairness. In line with the current findings, children could equally allocate resources when asked what they should give or how to allocate resources between two other people (Olson and Spelke, 2008; Smith et al., 2013; Rizzo and Killen, 2016). Interestingly, as third parties, children behaved more fairly and had stronger aversion to vicarious advantageous inequity than adults. The differences between age groups could be accounted for by lower ability of mentalizing among children than adults (Fehlbaum et al., 2022). In the third-party condition, participants acting as agents were asked to make allocation decisions for another dictator, which inherently requires for taking the perspectives from or empathizing with the represented dictator. In this regard, the ‘hyper-fair’ decisions in the role of third-party may reflect that children had more difficulty in placing themselves in the shoes of the represented partner. Another account of children’s third-party hyper-fairness is hypocrisy, that is, a desire to appear moral while avoiding the costs of being moral (Batson et al., 1999). In accordance, previous studies have observed hypocrisy behaviors in 6- to 8-year-old children (Shaw et al., 2014). According to this account, children might want to appear fair and disguise their unfairness in the second-party condition by acting hyper-fairly when deciding for others.

When deciding for oneself, adults still showed aversion to both advantageous and disadvantageous inequity, closely resembling their behaviors acting as third-party. These findings fit well with the inequity aversion model and behavioral control theory as well as a large body of empirical evidence on equity preferences. For instance, people are willing to achieve equity by rejecting not only disadvantageous but also advantageous offers from others, at the expense of their own payoffs (Hennig-Schmidt et al., 2008; Blake et al., 2015a). Likewise, people as dictators generously give about 30% of their endowment to passive recipients, instead of giving nothing to maximize their own payoffs (Engel, 2011). Moreover, equal outcomes compared to both disadvantageous and advantageous inequity induce brain regions important in reward processing, including ventral striatum and ventromedial prefrontal cortex (Tricomi et al., 2010; Zaki and Mitchell, 2011; Dawes et al., 2012). In contrast, behaviors leading to advantageous inequity were accompanied by the engagement of the anterior insula, a region previously associated with subjective disutility (Zaki and Mitchell, 2011). This is also consistent with the theory of behavioral control. Adults have internalized the behavioral control, they proactively activated the behavioral control in the first-party to inhibit the motivation for self-interest, resulting in the lack of a gap between behavior and knowledge. In light of previous findings, the current results suggest that adults do not only understand fairness knowledge but also show intrinsic preferences to fairness norms, such that they implement the acquired fairness knowledge even when their own interests are involved. However, it should be noted that adults may also exhibit advantage-seeking behaviors/preferences in some contexts, such as in tasks where they compete with others (Fliessbach et al., 2007, 2012).

Similar to adults, children also exhibited aversion to disadvantageous inequity. In line with the current findings, it has been demonstrated that children reject offers in which they receive less than others (McAuliffe et al., 2014). Moreover, aversion to disadvantageous inequity is not unique to humans, but instead previous studies have revealed evidence on the aversion across various species (Brosnan and de Waal, 2014; Blake et al., 2015a). Therefore, aversion to disadvantageous inequity might be shared among multiple species, which appears to have evolved as a fundamental mechanism of protecting personal interests and emerges/develops at a young age. In contrast to adults, children did not show aversion to advantageous inequity but instead exhibited advantage-seeking preferences. These findings cannot be accounted for by the inequity aversion model and behavioral control theory but are in line with the preferences for downward comparisons extensively established in the social psychology literature (Sheskin et al., 2014; Liu et al., 2017). That is, individuals exhibit positive emotions and reduce anxiety when they are better than others (Amoroso and Walters, 1969; Gibbons, 1986; Jankowski and Takahashi, 2014). Accordingly, children may act spitefully in certain contexts by choosing the advantageous option with personal cost over the equal option to maintain their superiority (Sheskin et al., 2014; Liu et al., 2017). These findings suggest that advantage-seeking represents an important motivation of resource allocation among children. Taken together, children exhibited the gap between fairness knowledge and behavior, while adults who have internalized behavioral control did not exhibit this gap, which is consistent with the theory of behavioral control.

The apparently different preferences/aversion for advantageous and disadvantageous inequity among children could be attributed to distinct neuropsychological processes underlying the two types of social preferences. On one hand, aversion to disadvantageous inequity appears to be driven mainly by negative emotions (Sanfey et al., 2003; Aoki et al., 2015; Feng et al., 2015; Gao et al., 2018), has been observed in numerous species (Brosnan and de Waal, 2014; Blake et al., 2015a), and occurs during early childhood in humans (McAuliffe et al., 2014). On the other hand, aversion to advantageous inequity seems to engage more complex social cognitive processes (Buckholtz and Marois, 2012; Li et al., 2014), and has been only observed in primates or humans (Riedl et al., 2012; Krueger and Hoffman, 2016), and manifests as children acquire social norms and develop social cognitive and behavioral control abilities (McAuliffe et al., 2015, 2017).

Children exhibited different preferences across tasks could attribute to both external and internal factors. The external factor is the competitiveness of the experimental context, where a competitive context increases the motivation for advantage-seeking. For example, even children as young as 9 and 10 years of age exhibited a willingness to choose the option favoring their own advantage (while reducing both their own and their partner’s payoff) over the option maximizing their absolute payoff (while benefiting their partner) to maintain their superiority (Herrmann et al., 2019). Furthermore, another evidence showed that the competitive context elicits envy and schadenfreude, which could induce the motivation for advantage-seeking in children (Steinbeis and Singer, 2013; Mendes et al., 2018). In contrast, when their prosocial emotion, such as empathy, was elicited, which would induce the motivation for equity in ultimatum game (Zheng et al., 2017; He et al., 2022). On the other hand, the internal factor is the level of internalization of behavioral control. As children age and behavioral control internalizes, the tendency for advantage-seeking diminishes, and aversion to inequity increases in allocation games (Fehr et al., 2013; Steinbeis and Singer, 2013; McAuliffe et al., 2014; Sheskin et al., 2014; McAuliffe et al., 2017).

Lastly, the current study did not identify the effects of loss/gain frame on fairness-related decision-making among either children or adults. These findings contradict previous observations and could be attributed to several reasons. First, it has been proposed that loss frame (vs. gain frame) exacerbates intuitive reactions in response to the conflict between self-interest and prosocial preferences (Slovic et al., 2007; Feng et al., 2021). However, recent evidence has indicated that the effects of loss/gain frame are diminished by the reminder of the payoff resulting from one’s decisions, since the manipulation might increase the reason and decrease the intuition in decision-making (Thunström, 2019). In the current study, participants were also informed of the payoffs resulting from their decisions, such that the final payoffs for both parties were displayed in real-time during decision-making. Therefore, children’s behavioral control might not be weakened and adults’ internalized behavioral control might not be enhanced in loss context. Second, the effects of loss/gain frame on fairness-related behaviors/preferences depend on individual variations in social value orientations, such that loss contexts promote prosocial individuals’ altruistic preferences but curtail individualists’ altruistic concerns (Dreu and McCusker, 1997; Reinders Folmer and De Cremer, 2012). In this regard, the null effects of loss/gain frame could be attributed to the reason that both prosocials and proselfs might have been recruited in the current study. Taken together, the null results of framing effect, which are inconsistent with behavioral control theory, could be attributed to the experimental design and participant recruitment.

Current study partially supported that behavioral control might play a crucial role in closing the gap between fairness knowledge and behavior. Additionally, our results found that children were not simply involved in the conflict between fairness norms and self-interest. Instead, children have another fundamental motivation for advantage-seeking. Therefore, children’s fairness decision-making is a complex outcome resulting from the interaction of multiple motivations. The understanding of this additional motivation might update the assumption regarding children’s allocation motivations in previous theory, and provide a possible way for further exploration into spiteful allocation (Fehr et al., 2008, 2013).

This work has several limitations, which raise important questions for future research. First, the selfish and advantage-seeking motivations could not be distinguished at behavioral level, as both motivations could drive participants to allocate more gains/less losses to themselves and expand advantageous inequity. Future studies may address this issue by including the conflicts between advantage-seeking and self-interest. Second, the current study only recruited two age groups, making the development trajectory of various fairness-related preferences unclear. Future studies could expand the age ranges to address the issue.

In summary, our study employed economic games and computational modeling to investigate the impact of framing on fairness-related knowledge and behavior as well as their underlying social preferences, and to explore the developmental direction of children’s fairness-related preferences. Our findings demonstrated that both children and adults exhibited similar behavioral pattern in third-party, exhibiting aversion to advantageous and disadvantageous inequity. However, they exhibited distinct behavioral patterns in first-party, such that both children and adults were averse to disadvantageous inequity, while children exhibited advantage-seeking preferences and adults exhibited aversion to advantageous inequity. These findings deepen our understanding of children’s social preferences and their developmental directions and have implications for institutional designs to promote the development of children’s prosocial behaviors, serving as a reminder to educators that multiple motives drive children’s prosocial behaviors or norm deviations.

## Data availability statement

The datasets presented in this study can be found in online repositories. The data and the analysis code are available at https://github.com/xuhan99/Common-and-distinct-equity-preferences-in-children-and-adults.git.

## Ethics statement

The studies involving humans were approved by the Ethics Committee of South China Normal University. The studies were conducted in accordance with the local legislation and institutional requirements. Written informed consent for participation in this study was provided by the participants’ legal guardians/next of kin.

## Author contributions

HX: Conceptualization, Data curation, Formal analysis, Writing – original draft, Writing – review & editing. LL: Writing – review & editing. RZ: Writing – review & editing. YuZ: Writing – review & editing. LZ: Writing – review & editing. YaZ: Writing – review & editing. CF: Conceptualization, Formal analysis, Funding acquisition, Project administration, Writing – original draft, Writing – review & editing. QG: Funding acquisition, Project administration, Supervision, Writing – original draft, Writing – review & editing.
