# Supplementary material for: Common and distinct equity preferences in children and adults
Source: Front Psychol. 2024 Feb 14;15:1330024. doi: 10.3389/fpsyg.2024.1330024 (PMC10899522; doi:10.3389/fpsyg.2024.1330024)
Supplement: Supplementary file 1 [file Table_1.DOCX]

Supplementary Material

# Behavioral Results

**TABLE S1** *
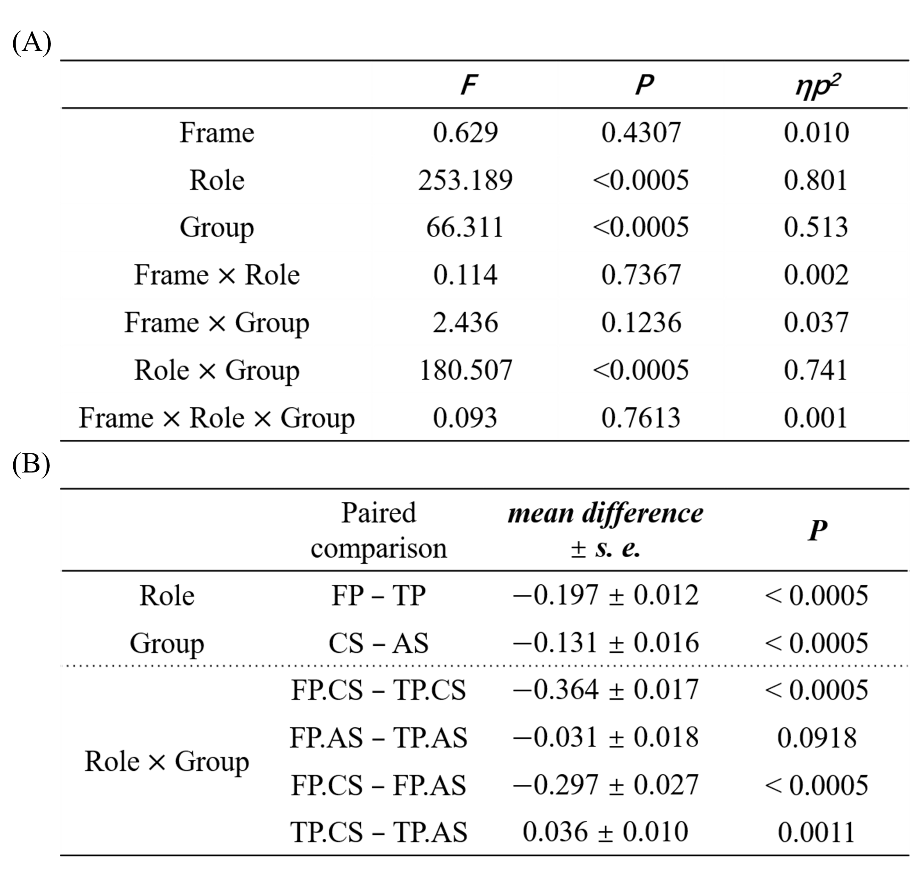
*A full list of statistical results of selfish deviation. (A) All main effects and interaction results. (B) Results of paired comparisons for significant effects.

*Note.* FP, first-party; TP, third-party; CS, children; AS, adults.

**TABLE S2**
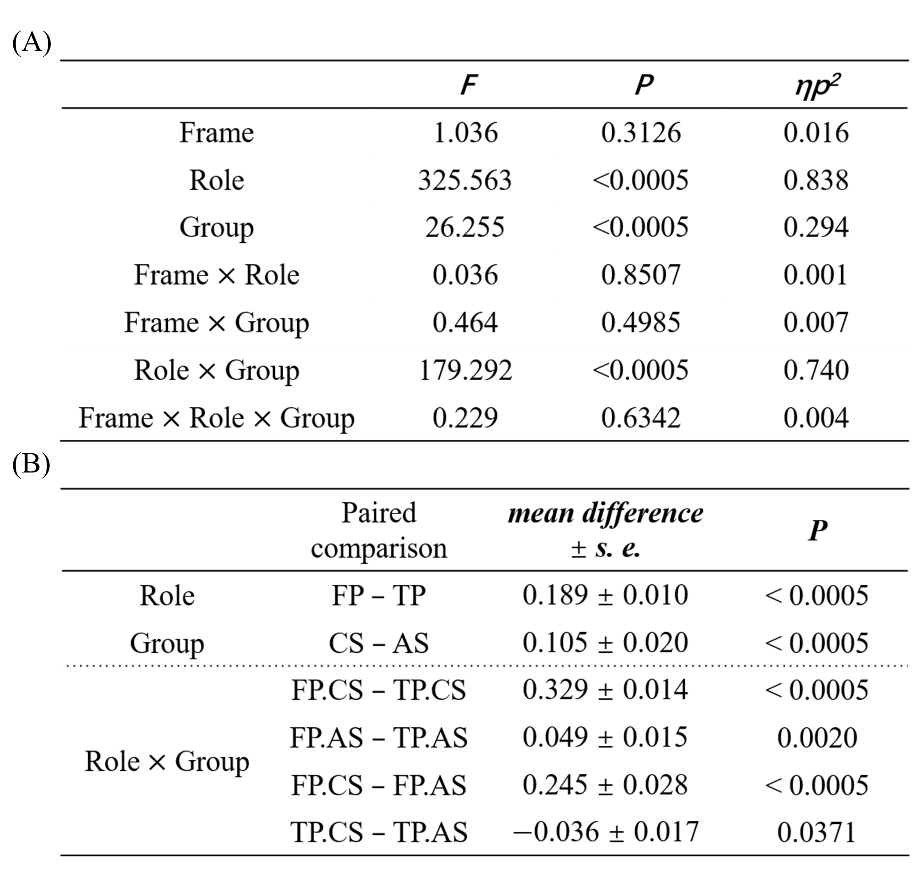
A full list of statistical results of equal deviation. (A) All main effects and interaction results. (B) Results of paired comparisons for significant effects.

*Note.* FP, first-party; TP, third-party; CS, children; AS, adults.

# Computational Results


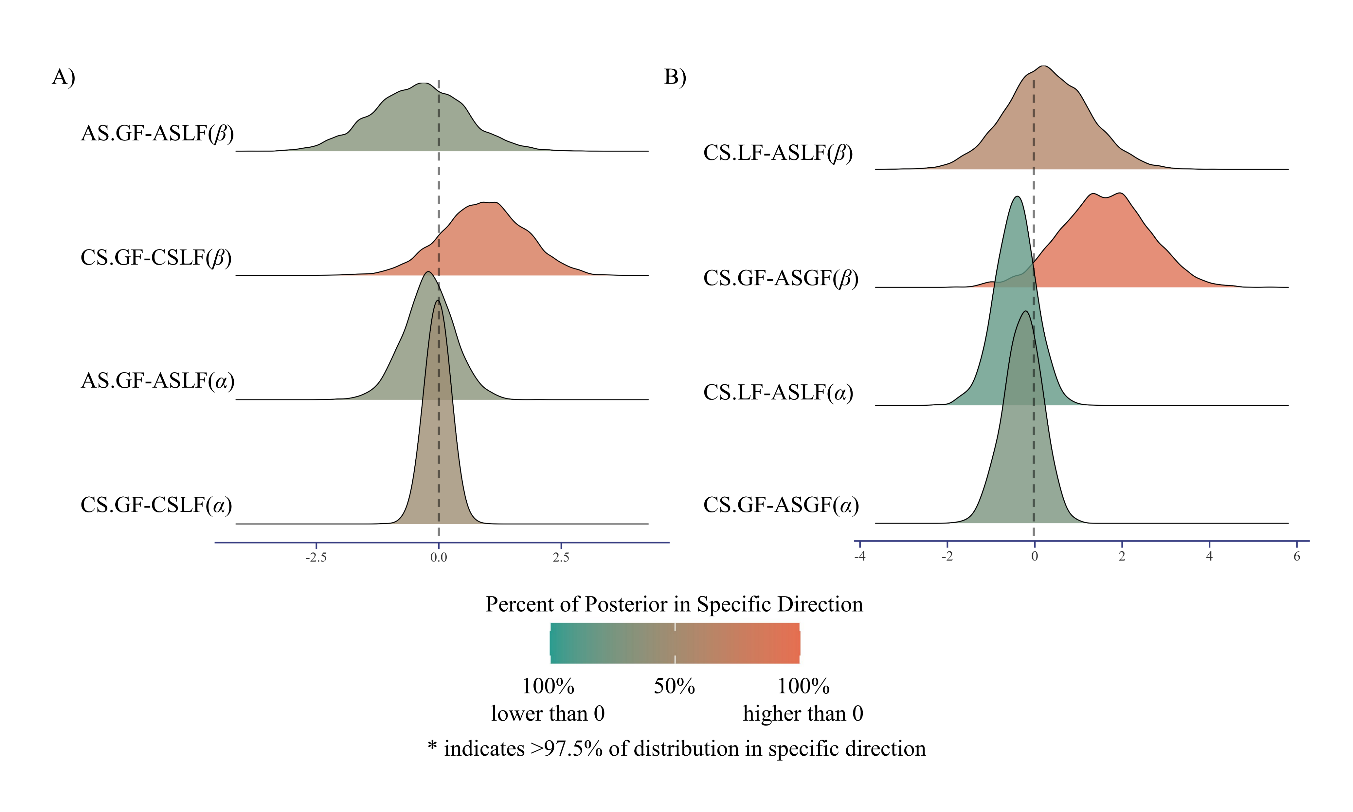


**FIGURE S1 | Frame results of group-level parameters. (A)** The effect of Frame. Children’s and adults’ parameters of aversion to advantageous (*α*) and disadvantageous (*β*) inequity showed no significant difference between frames (children (*α*), mean = -0.028, 95% HDI: [-0.537,0.478]; adult (*α*) mean = -0.202, 95% HDI: [-1.187,0.870]; children (*β*), mean = 0.900, 95% HDI: [-0.734,2.579]; adult (*β*), mean = -0.417, 95% HDI: [-2.350,1.315]). **(B)** The effect of Group. Parameters of aversion to advantageous (*α*) and disadvantageous (*β*) inequity in gain and loss frame showed no significant difference between groups (gain frame (*α*), mean = -0.274, 95% HDI: [-1.123,0.590]; loss frame (*α*), mean = -0.448, 95% HDI: [-1.371,0.450]; gain frame (*β*), mean = 1.601, 95% HDI: [-0.259,3.620]; loss frame (*β*), mean = 0.284, 95% HDI: [-1.634,2.121]). The shading and asterisks indicating the percentage of samples from the posterior greater than or less than 0. CS, children; AS, adults; GF, gain frame; LF, loss frame.


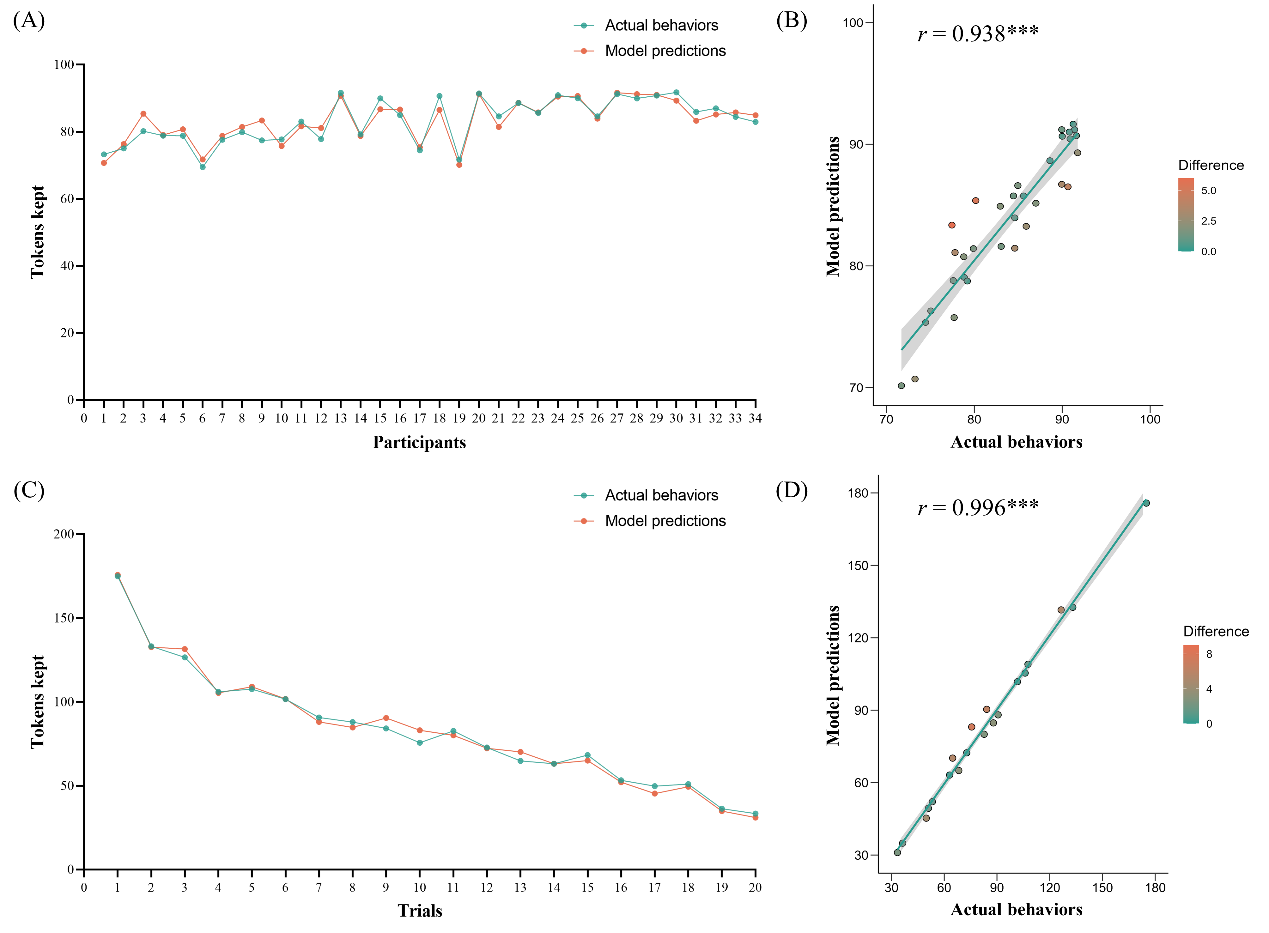


**FIGURE S2 | Model validations in children group, first-party role and gain frame. (A)** The average coins participants kept across 34 participants from actual behaviors and model predictions. The green line indicated actual behaviors observed, while the orange line indicated predictions of winning model (Model 4). **(B)** Correlation between actual behaviors and model predictions across 34 participants. The absolute differences between actual behaviors and model predictions were indicated by colors of dots from the largest (orange) to the smallest (green). **(C)** The average coins participants kept across 20 trials, the sequence of the trials had been rearranged based on experimental conditions. **(D)** Correlation and differences between actual behaviors and model predictions across 20 trials.


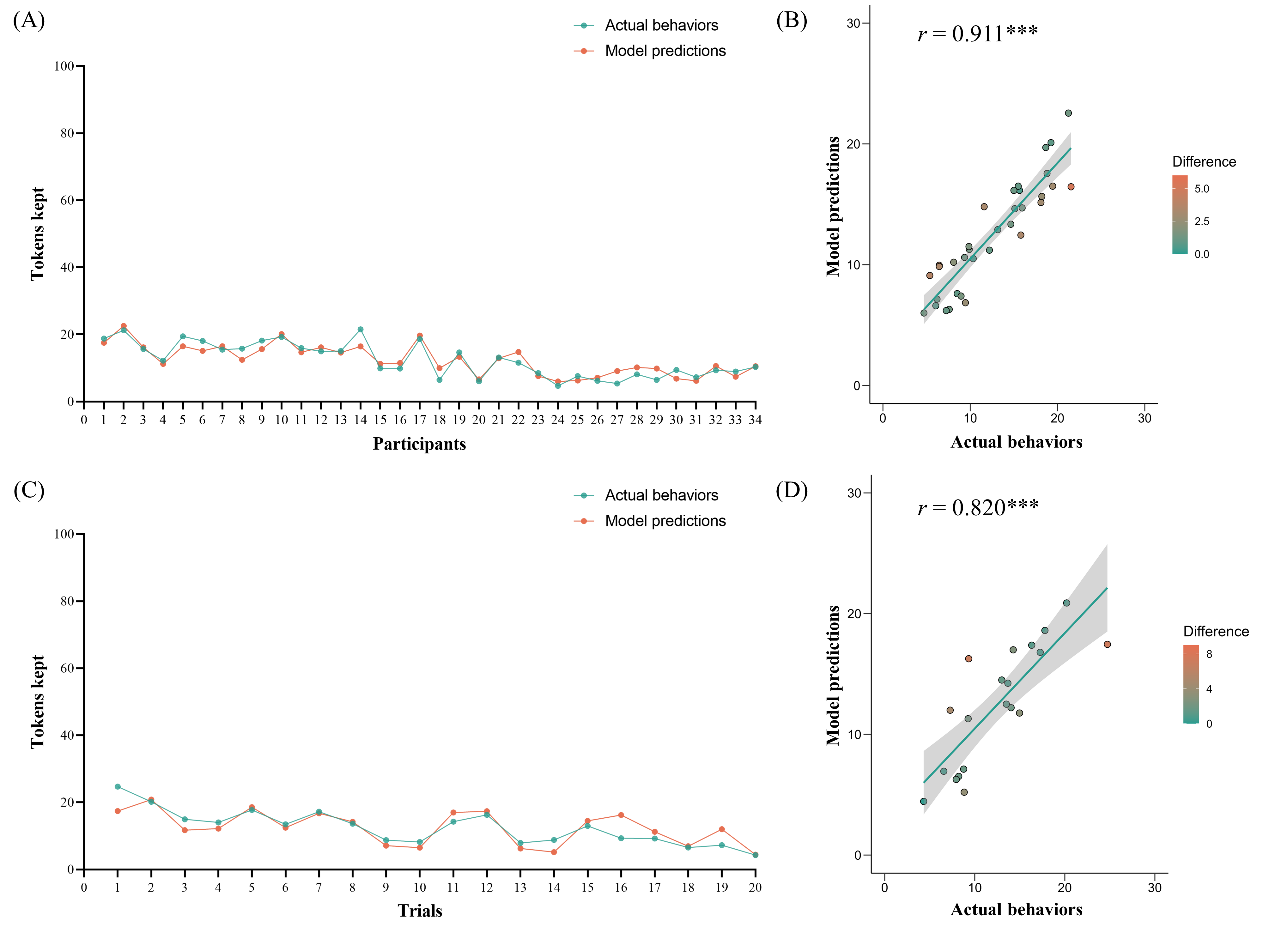


**FIGURE S3 | Model validations in children group, first-party role and loss frame. (A)** The average coins participants kept across 34 participants from actual behaviors and model predictions. The green line indicated actual behaviors observed, while the orange line indicated predictions of winning model (Model 4). **(B)** Correlation between actual behaviors and model predictions across 34 participants. The absolute differences between actual behaviors and model predictions were indicated by colors of dots from the largest (orange) to the smallest (green). **(C)** The average coins participants kept across 20 trials, the sequence of the trials had been rearranged based on experimental conditions. **(D)** Correlation and differences between actual behaviors and model predictions across 20 trials.


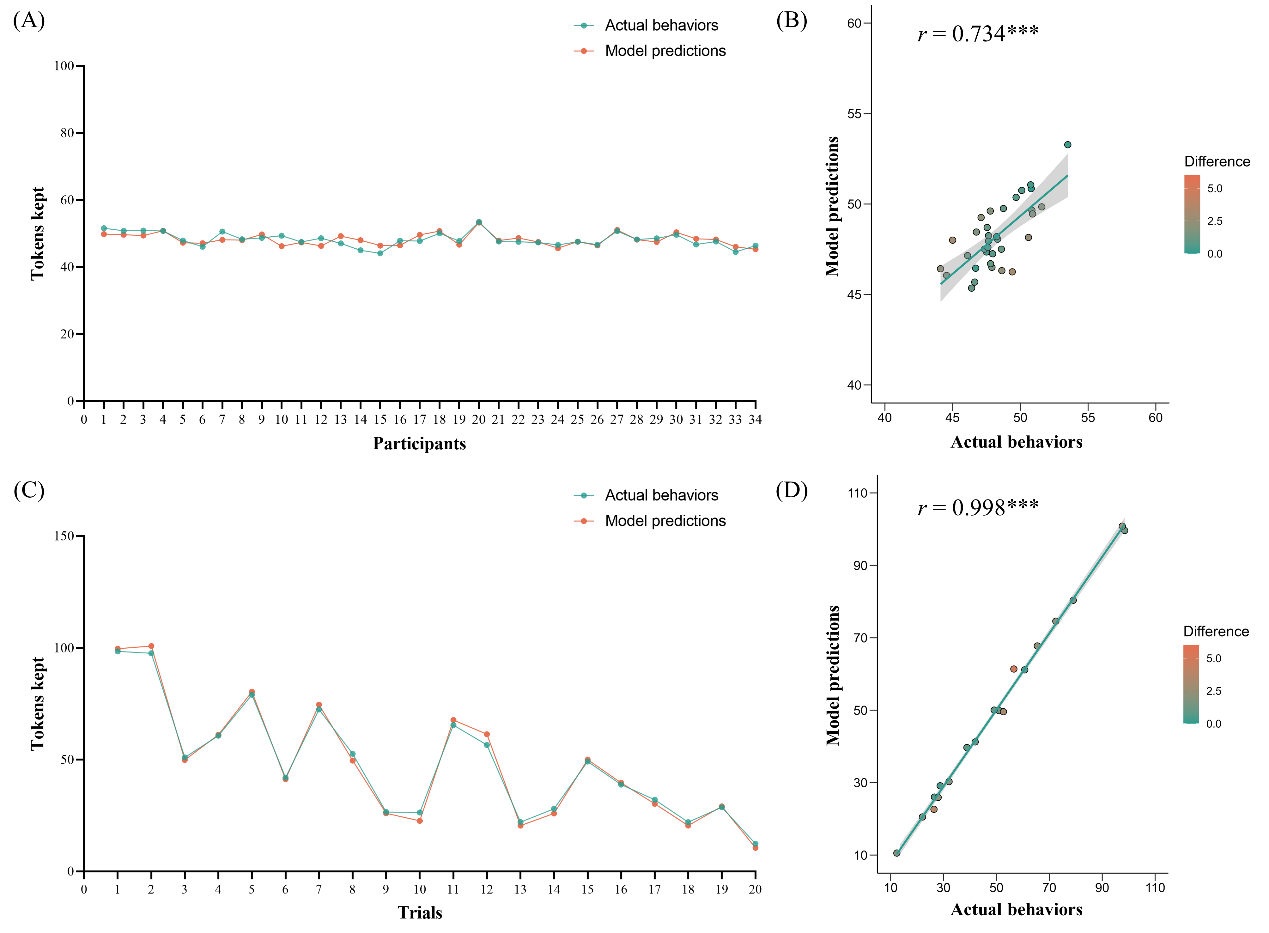


**FIGURE S4 | Model validations in children group, third-party role and gain frame. (A)** The average coins participants kept across 34 participants from actual behaviors and model predictions. The green line indicated actual behaviors observed, while the orange line indicated predictions of winning model (Model 4). **(B)** Correlation between actual behaviors and model predictions across 34 participants. The absolute differences between actual behaviors and model predictions were indicated by colors of dots from the largest (orange) to the smallest (green). **(C)** The average coins participants kept across 20 trials, the sequence of the trials had been rearranged based on experimental conditions. **(D)** Correlation and differences between actual behaviors and model predictions across 20 trials.


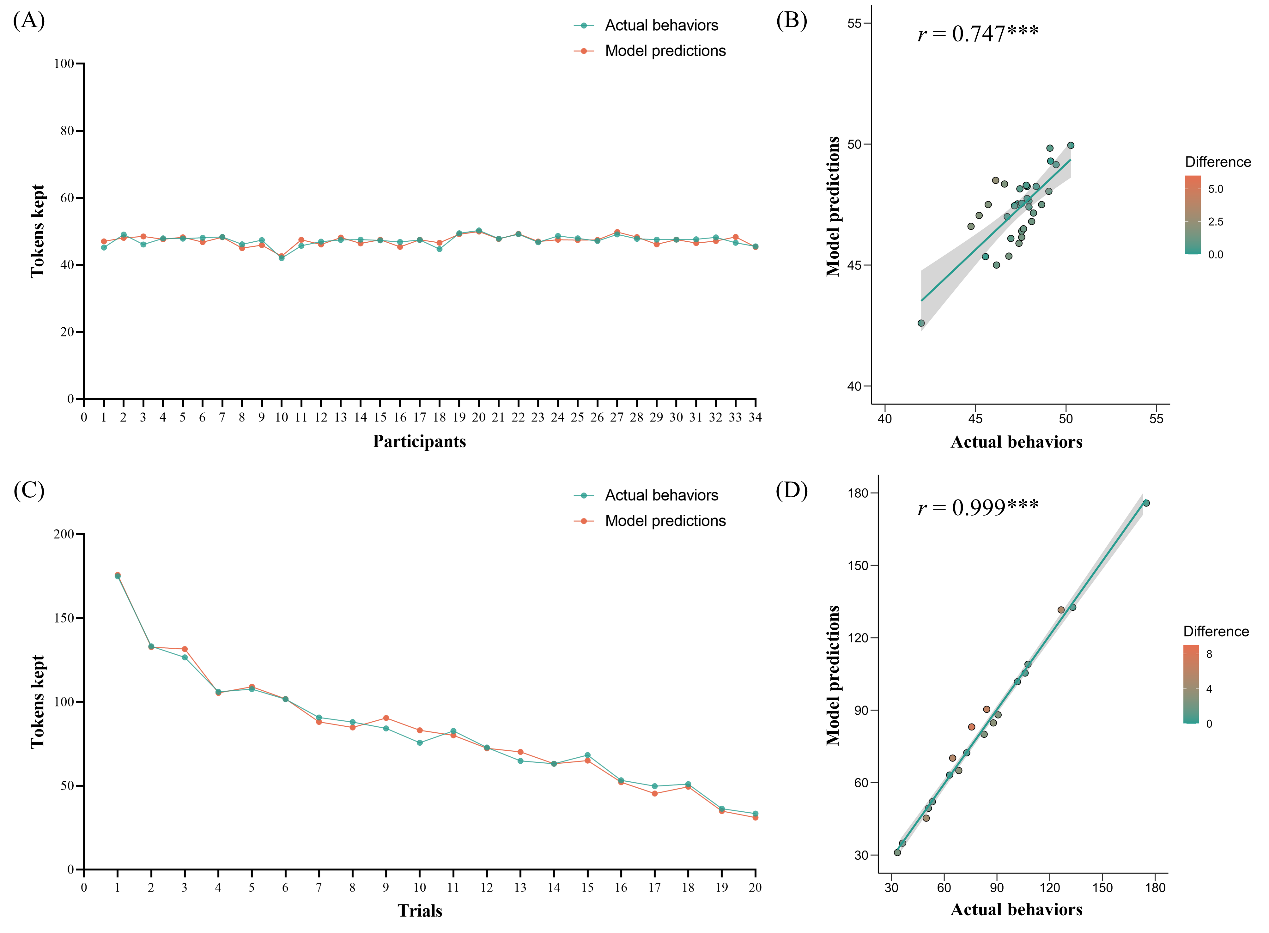


**FIGURE S5 | Model validations in children group, third-party role and loss frame. (A)** The average coins participants kept across 34 participants from actual behaviors and model predictions. The green line indicated actual behaviors observed, while the orange line indicated predictions of winning model (Model 4). **(B)** Correlation between actual behaviors and model predictions across 34 participants. The absolute differences between actual behaviors and model predictions were indicated by colors of dots from the largest (orange) to the smallest (green). **(C)** The average coins participants kept across 20 trials, the sequence of the trials had been rearranged based on experimental conditions. **(D)** Correlation and differences between actual behaviors and model predictions across 20 trials.


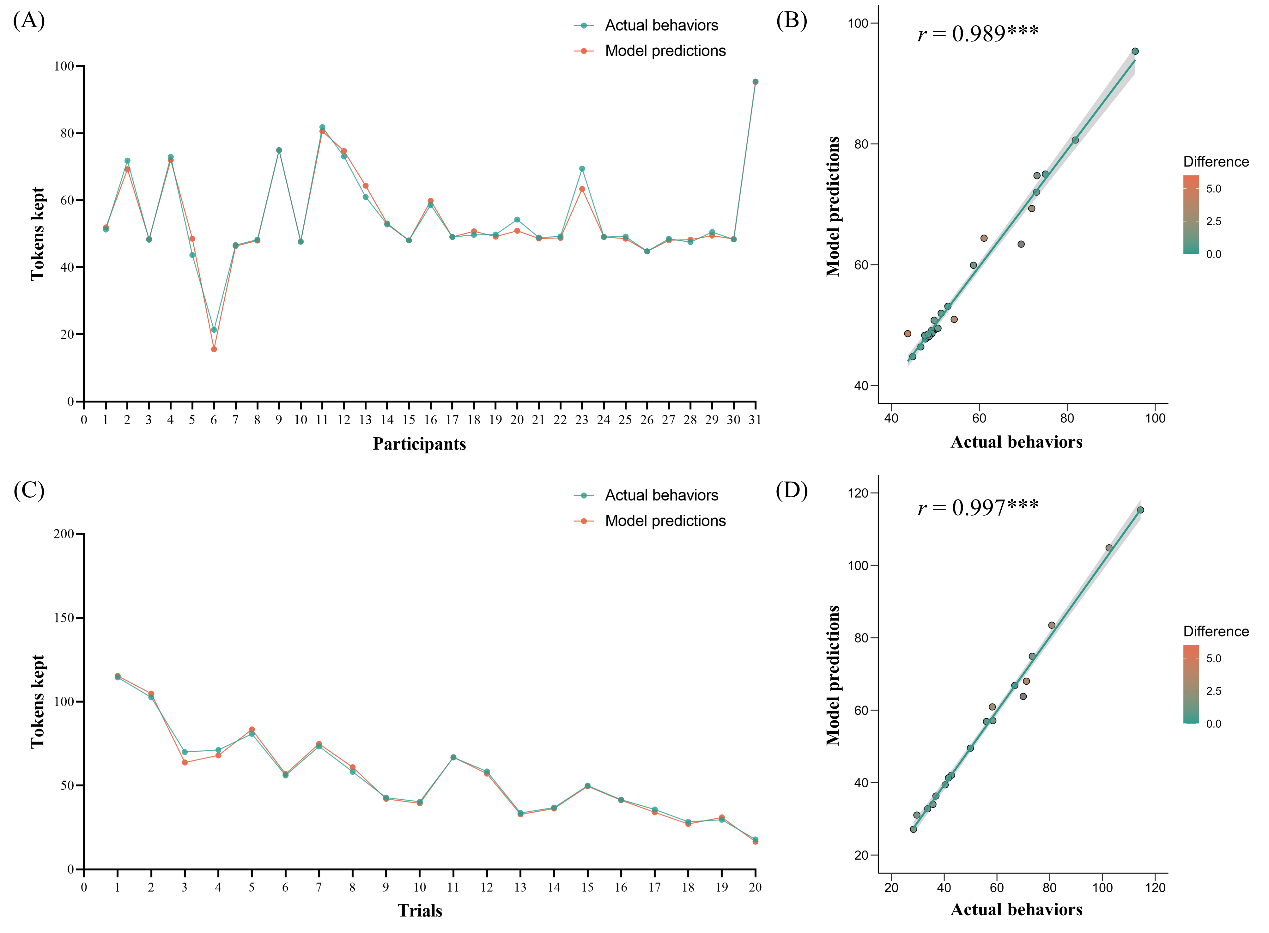


**FIGURE S6 | Model validations in adult group, first-party role and gain frame. (A)** The average coins participants kept across 31 participants from actual behaviors and model predictions. The green line indicated actual behaviors observed, while the orange line indicated predictions of winning model (Model 4). **(B)** Correlation between actual behaviors and model predictions across 31 participants. The absolute differences between actual behaviors and model predictions were indicated by colors of dots from the largest (orange) to the smallest (green). **(C)** The average coins participants kept across 20 trials, the sequence of the trials had been rearranged based on experimental conditions. **(D)** Correlation and differences between actual behaviors and model predictions across 20 trials.


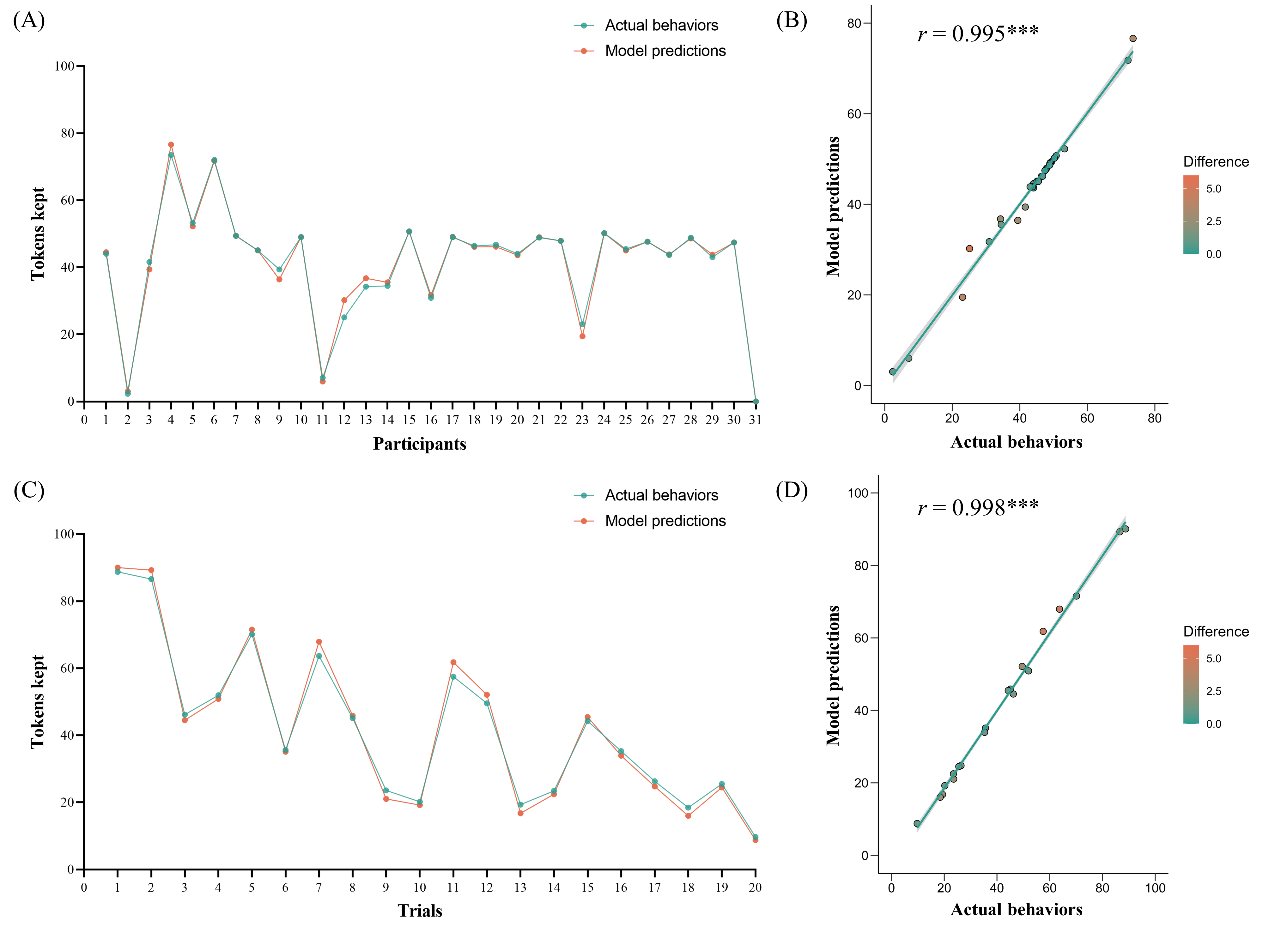


**FIGURE S7 | Model validations in adult group, first-party role and loss frame. (A)** The average coins participants kept across 31 participants from actual behaviors and model predictions. The green line indicated actual behaviors observed, while the orange line indicated predictions of winning model (Model 4). **(B)** Correlation between actual behaviors and model predictions across 31 participants. The absolute differences between actual behaviors and model predictions were indicated by colors of dots from the largest (orange) to the smallest (green). **(C)** The average coins participants kept across 20 trials, the sequence of the trials had been rearranged based on experimental conditions. **(D)** Correlation and differences between actual behaviors and model predictions across 20 trials.


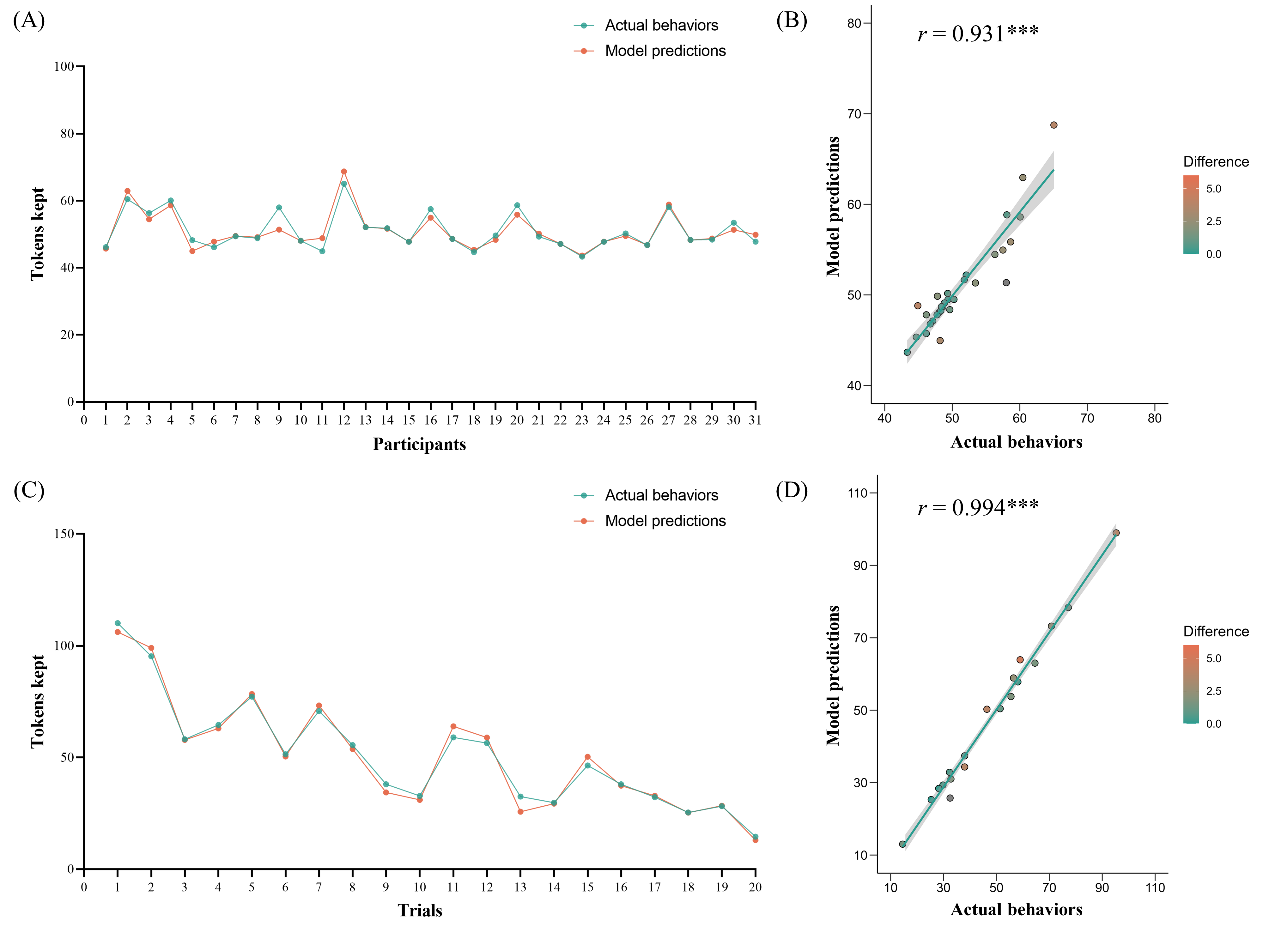


**FIGURE S8 | Model validations in adult group, third-party role and gain frame. (A)** The average coins participants kept across 31 participants from actual behaviors and model predictions. The green line indicated actual behaviors observed, while the orange line indicated predictions of winning model (Model 4). **(B)** Correlation between actual behaviors and model predictions across 31 participants. The absolute differences between actual behaviors and model predictions were indicated by colors of dots from the largest (orange) to the smallest (green). **(C)** The average coins participants kept across 20 trials, the sequence of the trials had been rearranged based on experimental conditions. **(D)** Correlation and differences between actual behaviors and model predictions across 20 trials.


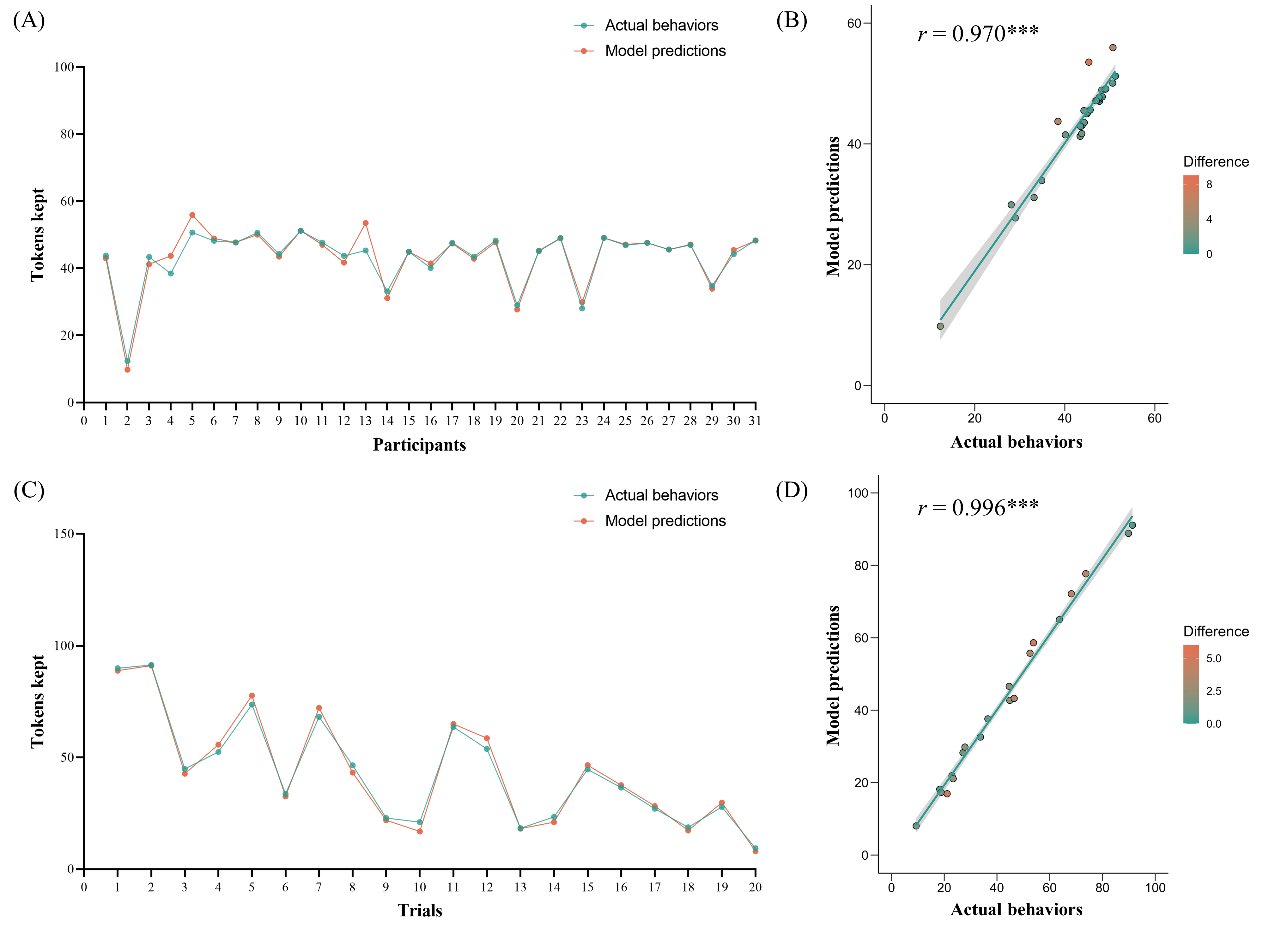


**FIGURE S9 | Model validations in adult group, third-party role and loss frame. (A)** The average coins participants kept across 31 participants from actual behaviors and model predictions. The green line indicated actual behaviors observed, while the orange line indicated predictions of winning model (Model 4). **(B)** Correlation between actual behaviors and model predictions across 31 participants. The absolute differences between actual behaviors and model predictions were indicated by colors of dots from the largest (orange) to the smallest (green). **(C)** The average coins participants kept across 20 trials, the sequence of the trials had been rearranged based on experimental conditions. **(D)** Correlation and differences between actual behaviors and model predictions across 20 trials.
